# Endothelial PD‐1 Regulates Vascular Homeostasis and Oligodendrogenesis during Brain Development

**DOI:** 10.1002/advs.202417410

**Published:** 2025-02-27

**Authors:** Tingting He, Mengtian Zhang, Jie Qin, Yanyan Wang, Sihan Li, Chaoyi Du, Jianwei Jiao, Fen Ji

**Affiliations:** ^1^ State Key Laboratory of Organ Regeneration and Reconstruction，Institute of Zoology Chinese Academy of Sciences Beijing 100101 China; ^2^ University of Chinese Academy of Sciences Beijing 100049 China; ^3^ Sino‐Danish College University of Chinese Academy of Sciences Beijing 100190 China; ^4^ Beijing Institute for Stem Cell and Regenerative Medicine, Institute for Stem Cell and Regeneration Chinese Academy of Sciences Beijing 100101 China

**Keywords:** angiogenesis, brain development, endothelial cells, oligodendrogenesis, PD‐1

## Abstract

Appropriate vascular and neural development is essential for central nervous system (CNS). Although programmed cell death receptor 1 (PD‐1) mediates neurogenesis, its role in cerebrovascular development remains poorly understood. Here, a correlation between cerebral vessels and oligodendrocyte precursor cells (OPCs) is revealed during brain development. The ablation of endothelial PD‐1 triggers cortical hypervascularization through excessive angiogenic sprouting, concomitantly driving OPC differentiation. These alterations disrupt blood brain barrier (BBB) maturation, induce dysmyelination, and ultimately result in abnormal behavior in mice. Mechanistically, the loss of endothelial PD‐1 suppresses the activity of the Wnt/β‐catenin signaling pathway, thereby disrupting normal angiogenesis. Concurrently, it activates the MEK1/2‐ERK1/2‐GLI1 pathway, leading to increased GREMLIN1 (GREM1) expression. Elevated GREM1 secretion inhibits the BMP/SMAD1/5/SMAD4 signaling cascade in OPCs, which inhibits oligodendrogenesis and myelination. These findings indicate the importance of endothelial cell‐intrinsic PD‐1 in regulating the oligovascular niche, and suggest potential therapeutic implications for neurological disorders associated with disrupted vascular development.

## Graphical abstract

Vascular endothelial cells are fundamental for angiogenesis and brain development. This study reveals that endothelial programmed cell death 1 (PD‐1) is critical for cerebral angiogenesis and blood brain barrier (BBB) maturation. It also mediates interaction with oligodendrocyte precursor cells (OPCs), modulating OPCs differentiation and myelination, offering therapeutic insights for neurological disorders.

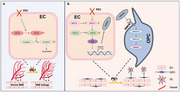

## 1 Introduction

Mouse cortical angiogenesis begins on embryonic day 8.5 (E8.5) with perineural plexus formation. By E9, the pial vessels penetrate the parenchyma, followed by ventral periventricular capillary sprouts extending dorsally at E10 to integrate with the pial vasculature, completing the primitive vascular network.^[^ ^1^ ^]^ The development of cerebral blood vessels is essential for establishing the blood brain barrier (BBB), which is a specialized protective interface between the circulatory and neural systems.^[^ ^2^, ^3^ ^]^ The BBB is a complex and dynamic structure formed not only by tightly sealed brain capillary endothelial cells but also by interactions with pericytes, astrocytes, and the extracellular matrix, collectively known as the neurovascular unit.^[^ ^4^, ^5^ ^]^ Pericytes envelop endothelial cells and contribute to BBB integrity by regulating endothelial tight junctions and vascular stability,^[^ ^6^ ^]^ while astrocyte end‐feet ensheath the capillaries, influencing BBB permeability and facilitating nutrient transport.^[^ ^7^ ^]^ To its structural roles, the BBB regulates nutrient delivery, waste clearance, and immune cell trafficking, which are critical for brain homeostasis and neuroprotection.^[^ ^8^ ^]^

Brain vascular networks dynamically interact with neural stem cells and glial progenitor cells during development, orchestrating coordinated neurogenesis and gliogenesis.^[^ ^9^ ^]^ Vasculature forms the special microenvironment, called the “neurovascular niche,” secreting growth factors, cytokines or just providing a physical supportive structure to regulate neural stem cell proliferation and differentiation.^[^ ^10^, ^11^, ^12^ ^]^ Astrocytes also form direct contact with cerebral blood vessels. Vasculature‐derived signals such as Ascl1 can influence astrocyte fate determination, and in turn, astrocytes are indispensable for maintaining BBB integrity and regulating vessel diameter.^[^ ^13^, ^14^, ^15^ ^]^ Oligodendrogenesis is a complex process that involves a wide range of mechanisms, including neuronal activity, astrocyte‐derived signals, extracellular matrix components, and intrinsic genetic programs.^[^ ^16^, ^17^ ^]^ The concept of the “oligovascular niche,” an extension of the neurovascular niche, has been proposed to explore the close relationship between cerebral vessels and oligodendrocyte precursor cells (OPCs).^[^ ^18^ ^]^ It is believed that endothelial cells can promote OPC survival and proliferation by secreting trophic factors such as VEGF, BDNF, and bFGF.^[^ ^19^, ^20^ ^]^ Conversely, OPCs have been found to regulate vessel growth. For instance, OPCs interact with sprouting endothelial tip cells to regulate neonatal white matter vessel formation in a Wnt‐dependent manner.^[^ ^21^ ^]^ Dysfunction of these niches has been implicated in conditions such as autism, stroke, Alzheimer's disease, and other disorders.^[^ ^22^, ^23^, ^24^, ^25^ ^]^

Programmed cell death protein 1 (PD‐1), a major immune checkpoint, controls immune tolerance, activates immune responses when needed, and helps to build natural defenses against pathogens. PD‐1 is highly expressed in activated T cells and other immune cells, and regulates immune activity in both central nervous system‐associated and peripheral immune organs.^[^ ^26^ ^]^ While PD‐1/PD‐L1 (Programmed cell death ligand 1) is best known for controlling T‐cell immunity, new findings show that it directly regulates vascular endothelial cell functions. For example, the PD‐1/PD‐L1 signaling axis is essential for modulating the immune response to atherosclerosis.^[^ ^27^ ^]^ Utilization of PD‐1/PD‐L1 inhibitors can exacerbate atherosclerosis, thereby elevating the risk of myocardial infarction and stroke.^[^ ^28^ ^]^ Moreover, PD‐1 plays an essential role in the central nervous system. Loss of PD‐1 in neural stem cells disrupts neurogenesis by boosting neural progenitor proliferation and blocking their maturation, ultimately inducing depression‐like behavior in mice.^[^ ^29^ ^]^ The expression of PD‐1 in neurons has also been implicated in the regulation of acute and chronic pain.^[^ ^30^ ^]^ Suppression of PD‐1 expression mitigates the onset of pain. In addition, learning and memory are subject to the regulatory influence of neural PD1 signaling.^[^ ^31^ ^]^

However, the role of PD‐1 in endothelial cells during cerebrovascular development is not fully understood. Understanding PD‐1′s specific roles in endothelial cells will deepen insight into cerebral vasculature biology and brain development regulation, potentially offering new strategies for the treatment of neurovascular and neurodevelopmental disorders. In the present study, we found that endothelial PD‐1 regulates angiogenesis and BBB formation. Loss of endothelial PD‐1 promotes angiogenesis but impairs the endothelium tight junction and BBB integrity, mainly through the downregulation of the Wnt/β‐catenin signaling pathway. Furthermore, the deletion of endothelial PD‐1 facilitated the differentiation of OPCs into oligodendrocytes and myelination. We demonstrated that the loss of endothelial PD‐1 enhanced MEK1/2‐ERK1/2 signaling, which further upregulated endothelial GREMLIN1 (GREM1) secretion. GREM1 binds to BMPs and thus inhibits the OPCs’ BMP‐SMADs signaling pathway, promoting the differentiation of OPCs in the early postnatal period. In addition, abnormalities in the BBB and brain development result in abnormal behavior in mice, providing a perspective for the treatment of neurological disorders by targeting the cerebral endothelium.

## 2 Results

### 2.1 Endothelial PD‐1 Deletion Promotes Angiogenesis

**Figure 1:**
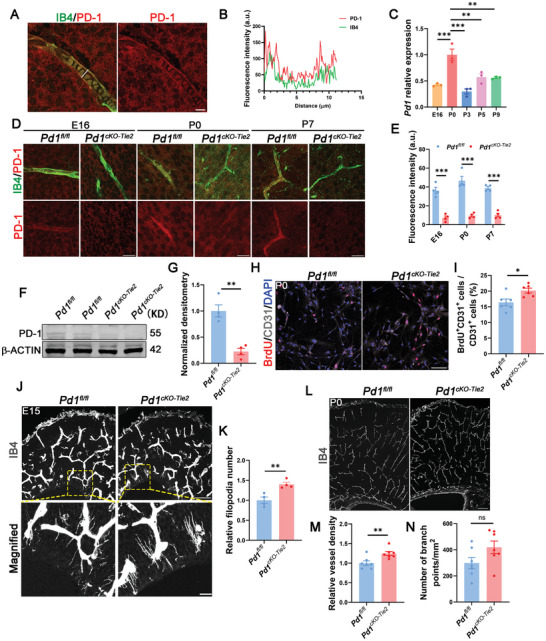
Endothelial PD‐1 ablation causes abnormal angiogenesis. A) Confocal immunofluorescence image of IB4^+^ and PD‐1^+^ vessels. Scale bar, 10 µm. B) Vascular co‐localization analysis of PD‐1 (red) and IB4 (green). The analysis area is the white line region in panel A. C) Relative *Pd1* mRNA levels of purified brain endothelial cells at E16, P0, P3, P5, and P9. *n* = 3 mice for each group, p = 0.0002 (E16), *p* < 0.0001 (P3), p = 0.0021 (P5), p = 0.0017 (P9), P0 group as reference group, one‐way ANOVA with Dunnett multiple comparison test. D) Immunofluorescence staining of vascular PD‐1 and IB4 in E16, P0, P7 mice brain slices. Scale bar, 20 µm. E) Fluorescence intensity analysis of vascular PD‐1 at E16, P0, P7. *n* = 4 mice for each group, p = 0.000279 (E16), p = 0.000232 (P0), *p* < 0.0001 (P7), multiple t tests, statistical significance determined using the Holm‐Sidak method, with alpha = 0.05. F) Western blot analysis of the expression level of PD‐1 in *Pd1^fl/fl^* and *Pd1^cKO‐Tie2^* mice endothelial cells, β‐ACTIN detected as loading control. G) Graph showed the normalized densitometry of PD‐1. *n* = 4 mice for each group, p = 0.0011, two‐tailed unpaired t test. H) Immunofluorescent staining of BrdU and CD31 in endothelial cells isolated from *Pd1^fl/fl^* and *Pd1^cKO‐Tie2^* mice brain. Scale bar, 100 µm. I) Proliferation capacity of *Pd1^cKO‐Tie2^* mice endothelial cells was improved. *n* = 6 mice for each group, p = 0.0175, two‐tailed unpaired t test. J) Confocal images of IB4^+^ vessels in *Pd1^fl/fl^* and *Pd1^cKO‐Tie2^* mice brain slices at E15. The lower panel showed the magnified image of the delineated area. Scale bar, 100 µm (upper), 20 µm (lower). K) Filopodia number analysis of *Pd1^fl/fl^* and *Pd1^cKO‐Tie2^* neocortex vessels at E15. *n* = 4 mice for each group and p = 0.006, two‐tailed unpaired t test. L) The density of brain vessels in P0 *Pd1^cKO‐Tie2^* mice was increased. Scale bar, 200 µm. M) Graph showed the relative vessel density. *n* = 7 mice for each group, p = 0.0062, two‐tailed unpaired t test. N) Graph showed the number of branch points. *n* = 7 mice for each group. Data are means and SEM, *p* < 0.05(*), *p* < 0.01(**), *p* < 0.001(***), ns not significant.

Endothelial cells are indispensable elements of blood vessels and are widely recognized to influence brain development.^[^ ^32^ ^]^ PD‐1 signaling is thought to be associated with angiogenesis, particularly in the tumor microenvironment, where the expression of PD‐1 may influence the generation and functionality of blood vessels.^[^ ^33^ ^]^ To verify the endothelial PD‐1 expression, immunofluorescence staining was performed on mouse brain slices and cultured endothelial cells. Both in vivo and in vitro experiments consistently demonstrated PD‐1 expression in vascular endothelial cells (**Figure** **1**A,B; Figure S1A, Supporting Information). We investigated temporal changes in endothelial PD‐1 expression during brain development by isolating cerebrovascular cells from E16 (embryonic day 16), P0 (postnatal day 0), P3 (postnatal day 3), P5 (postnatal day 5), P9 mice (Postnatal day 9), then detecting *Pd1* mRNA expression levels by RT‐qPCR analysis. The results showed that endothelial *Pd1* expression was the highest in the P0 period compared to the other stages (Figure 1C). Moreover, cerebral vessel staining revealed fluctuations in PD‐1 expression in endothelial cells, with a notable increase in fluorescence intensity observed at the P0 stage (Figure 1D). These results suggest that endothelial PD‐1 plays an important role in P0 during brain development.

To study endothelial PD‐1 function in central nervous system development, *Pd1^fl/fl^* mice were intercrossed with *Tie2‐Cre* mice, which facilitated efficient recombination, specifically in the endothelial cell lineage. This strategic mating resulted in the generation of endothelial cell‐specific conditional knockout mice (*Pd1^cKO‐Tie2^*), which helped us to study how the loss of PD‐1 in endothelial cells affects neural development (Figure S1B, Supporting Information). Allele‐specific PCR confirmed successful *Pd1* recombination in *Pd1^cKO‐Tie2^* mice. This genetic ablation led to concurrent decreases in both PD‐1 protein and *Pd1* mRNA, validating the endothelial‐specific knockout efficiency (Figure 1G; Figure S1C, Supporting Information). We also restricted PD‐1 knockout to endothelial bells by eliminating off‐target effects in other cell types (Figure S1D, Supporting Information).

Next, changes in the vascular morphology were examined after *Pd1* deletion. In vitro assays revealed that PD‐1 knockout endothelial cells exhibited markedly enhanced proliferation in conditioned medium, as shown by BrdU staining (Figure 1H,I). Translating these findings into vivo, the E15 cortical vasculature in *Pd1^cKO‐Tie2^* mice displayed excessive filopodial protrusions within the neurogenic niches (VZ/SVZ) (Figure 1J,K). By birth (P0), while the vascular branching complexity remained unchanged, cortical vessels showed a significant increase in density, suggesting sustained postnatal hypervascularization (Figure 1L,M). Molecular analysis corroborated these morphological changes, with upregulated expression of the endothelial markers CD31, VE‐cadherin, and VEGFR in knockout cortices (Figure S1G, Supporting Information). Importantly, these phenotypes were brain‐specific, as the peripheral organs showed no angiogenic alterations (Figure S1H–J, Supporting Information). Overall, these results suggested that endothelial *Pd1* deletion promotes angiogenesis during brain development.

### 2.2 Loss of Endothelial PD‐1 Affects Blood‐Brain Barrier Integrity

**Figure 2:**
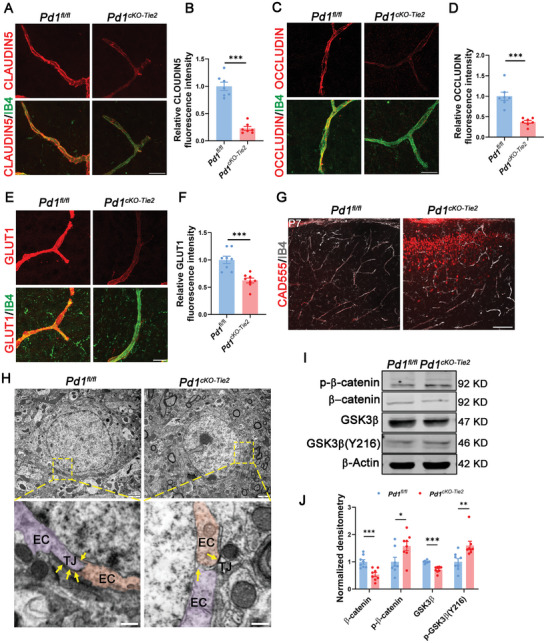
Endothelial PD‐1 deletion impairs the tight junction and blood brain barrier formation. A) CLAUDIN5 expression in *Pd1^cKO‐Tie2^* mice brain vessels was decreased. Scale bar, 20 µm. B) Graph showed the relative CLAUDIN5 fluorescent intensity of *Pd1^fl/fl^* and *Pd1^cKO‐Tie2^* mice vessels. *n* = 7 mice for each group, *p* < 0.0001, two‐tailed unpaired t test with Welch's correction. C) Confocal immunofluorescence images of OCCLUDIN and IB4 in *Pd1^fl/fl^* and *Pd1^cKO‐Tie2^* mice brain vessels. Scale bar, 20 µm. D) Graph showed the relative OCCLUDIN fluorescent intensity of *Pd1^fl/fl^* and *Pd1^cKO‐Tie2^* mice vessels. *n* = 7 mice for each group, p = 0.0004, two‐tailed unpaired t test with Welch's correction. E) GLUT1 expression was decreased in *Pd1^cKO‐Tie2^* mice brain vessel. Scale bar, 20 µm. F) Quantification of relative vessel's GLUT1 fluorescent intensity. *n* = 8 mice for each group, p = 0.0003, two‐tailed unpaired t test. G) Confocal images of CAD555 and IB4 in coronal brain slices of *Pd1^fl/fl^* and *Pd1^cKO‐Tie2^* mice at P7. Scale bar, 100 µm. H) Electron microscopy of *Pd1^fl/fl^* and *Pd1^cKO‐Tie2^* mice brain vessels. Right, high‐magnification views of the delineated area (EC: endothelial cell, TJ: tight junction). Scale bar, 2 µm (left), 500 nm (right). I) Western blot analysis of the expression levels of β‐catenin, p‐β‐catenin, GSK3β, GSK3β(Y216) of *Pd1^fl/fl^* and *Pd1^cKO‐Tie2^* mice endothelial cells, β‐ACTIN detected as loading control. J) Graph showed the relative expression levels of Wnt/β‐catenin pathway related proteins are decreased. *n* = 8 mice for each group, p = 0.000814 (β‐catenin), p = 0.027836 (p‐β‐catenin), *p* < 0.0001 (GSK3β), p = 0.007802 (p‐GSK3β(Y218)), multiple t tests, statistical significance determined using the Holm‐Sidak method, with alpha = 0.05. Data are means and SEM, *p* < 0.05(*), *p* < 0.01(**), *p* < 0.001(***), ns not significant.

Endothelial cells are a central part of the BBB, and neurovascular coupling regulates brain homeostasis. To determine whether endothelial PD‐1 deletion alters BBB integrity, we analyzed CLAUDIN5 expression, a central tight junction protein critical for the structural assembly of endothelial barriers and the regulation of BBB permeability.^[^ ^34^ ^]^ CLAUDIN5 and Isolectin B4 (IB4) double staining showed that CLAUDIN5 expression was significantly lower in *Pd1^cKO‐Tie2^* mice than in their wild‐type littermates (**Figure** **2**A,B). Similar to CLAUDIN5, OCCLUDIN is an integral membrane protein that contributes to BBB maintenance.^[^ ^35^ ^]^ There was no surprise that the *Pd1^cKO‐Tie2^* mice also showed less OCCLUDIN expression (Figure 2C,D). However, Zonula Occludens protein 1 (ZO‐1), a component of tight junctions, showed no significant difference between the two groups (Figure S2A,B, Supporting Information). These findings indicate that endothelial PD‐1 ablation selectively disrupts BBB integrity through targeted downregulation of key transmembrane tight junction components while sparing cytoplasmic plaque proteins such as ZO‐1. Collagen type IV is indispensable for the endothelial basement membrane, which is essential in early blood vessel morphogenesis and stability.^[^ ^36^ ^]^ Immunostaining showed that *Pd1^cKO‐Tie2^* mice had fewer collagen IV‐positive vessels than *Pd1^fl/fl^* mice (Figure S2C,D, Supporting Information). This indicates that PD‐1 ablation may impair cerebrovascular maturation by destabilizing the structural foundation of developing vessels. Recruitment of vascular pericytes was then evaluated, and no significant differences were observed between the two groups (Figure S2E–G, Supporting Information). Glucose transporter 1 (GLUT1), a key glucose transporter in the BBB, is expressed on the luminal surface of cerebrovascular endothelial cells. It facilitates glucose delivery to the brain, ensuring proper neuronal function and energy homeostasis.^[^ ^37^ ^]^ We performed GLUT1 fluorescent staining on P0 brain sections, and the results revealed that GLUT1 expression was significantly decreased in *Pd1^cKO‐Tie2^* mice compared with *Pd1^fl/fl^* mice, suggesting that PD‐1 ablation compromises cerebrovascular metabolic competence through impaired glucose transporter trafficking (Figure 2E,F).

We used a combination of methods to comprehensively evaluate the integrity of the BBB, we utilized a combination of methods. Alexa Fluor 555 cadaverine (CAD555, a 555 Da impermeable tracer) was intravenously administered. After a 2‐hour circulation period, significant extravasation of the tracer into the brain parenchyma was observed in *Pd1^cKO‐Tie2^* mice, indicating a compromised functional barrier (Figure 2G). Subsequently, to verify the structural basis of this compromise, we conducted transmission electron microscopy (TEM) of cerebrovascular endothelial cells. The results revealed that in contrast to the continuous junctional complexes observed in *Pd1^fl/f^* ^l^ control mice, knockout mice exhibited discontinuous tight junction strands (Figure 2H). The wnt/β‐catenin signal pathway is regarded as a key regulator in the development of the BBB. We detected the expression of several proteins related with Wnt/β‐catetnin (β‐catenin, p‐β‐catenin, GSK‐3β, GSK‐3β (Y216)) and revealed that endothelial PD‐1 ablation inhibits canonical Wnt signaling (Figure 2I–K). However, the BBB of the adult mice functioned normally (Figure S2I, Supporting Information). Overall, loss of endothelial PD‐1 caused endothelial tight junction homeostasis breakdown and BBB maturation impairment during early development, which may be caused by Wnt/β‐catenin cascade suppression.

### 2.3 Endothelial PD‐1 Loss does not Affect Neurogenesis and Astrogenesis but Oligodendrogenesis

Neural tube vasculogenesis is almost synchronized with neurogenesis and gliogenesis. To further reveal the function of endothelial cells in cortical development, we characterized the spatial distribution patterns of vascular networks, neural progenitor cells (NPCs), and glial precursor cells (GPCs). The results showed that the embryonic brain was extensively vascularized, and blood vessels were physically adjacent to NPCs and GPCs, suggesting that vasculogenesis is closely associated with neurogenesis and gliogenesis (Figure S3A,B, Supporting Information). We hypothesized that the endothelial PD‐1 knockout affects neurogenesis. We first evaluated the NPC self‐renewal capacity at E16 by quantifying KI67 positive and Phospho‐histone3 (PH3)‐positive proliferating cells through immunostaining. Quantitative comparisons revealed no significant differences in proliferation indices between *Pd1^cKO‐Tie2^* and *Pd1^fl/fl^* mice, indicating comparable NPC proliferative activity across genotypes at this developmental stage (Figure S3C–F, Supporting Information). Intermediate progenitors (IPs) are a major subclass of neural progenitor cells that are critical regulators of cortical neurogenesis. The immunostaining results showed that loss of endothelial PD‐1 did not alter the density of TBR2 (a marker of IPs) cells expression level, indicating preserved IP specification dynamics despite vascular abnormalities (Figure S3G,H, Supporting Information) To determine the impact of endothelial‐specific PD‐1 deletion on cortical layer specification, the layer V/VI specific marker CTIP2 was applied to further investigate the position of neuron. Immunofluorescence analysis of cortical sections and western blot quantification revealed that endothelial‐specific PD‐1 deletion failed to disrupt the laminar positioning of layer V/VI neurons, as evidenced by the preserved CTIP2^+^ cellular stratification patterns (Figure S3I–L, Supporting Information). In addition, layer II/III neurons showed no significant differences between the two groups (Figure S3K,L, Supporting Information). Loss of endothelial PD‐1 did not affect the localization or number of TBR1 positive immature neurons (Figure S3M,N, Supporting Information). Taken together, these results suggested that PD‐1 depletion did not affect neurogenesis or migration. In addition to neurogenesis, angiogenesis is also accompanied by astrogenesis. Quantitative GFAP immunofluorescence analysis demonstrated that endothelium‐specific PD‐1 deletion had no detectable influence on astrocyte generation (Figure S3O,P, Supporting Information). Methodological consistency was confirmed through western blot quantification of GFAP protein levels across cortical lysates, which revealed no intergroup differences in GFAP expression profiles (Figure S3Q,R, Supporting Information). Thus, endothelial depletion of PD‐1 did not affect cortical astrogenesis.

Although microglia do not originate from the cortical neuroepithelium, they colonize the brain around E9 in mice and subsequently execute critical regulatory functions. Dual immunofluorescence labeling of E13 neocortical sections with IB4 and CX3CR1 (microglial markers) revealed striking spatiotemporal coordination between the developing vasculature and microglial populations (Figure S3S, Supporting Information). We labeled CX3CR1^+^ microglia with GFP and found a significant increase in the proportion of CX3CR1‐GFP^+^ and CD68^+^ microglia within the cerebral cortex of *Pd1^cKO‐Tie2^* mice (Figure S3T,U, Supporting Information). This observation was further confirmed by western blotting and RT‐qPCR data, which showed that the ablation of endothelial cell PD‐1 led to the amplification of microglia (Figure S3V–X, Supporting Information). Additionally, we measured the expression levels of inflammation‐related molecules in the brain via RT‐qPCR, including IL‐1β, TNF‐α, IL‐6 (Figure S3Y, Supporting Information). The results showed a marked elevation in inflammatory markers in the brains of knockout mice compared with controls. In contrast, E13 specimens, corresponding to the pre‐vascularization stage, exhibited baseline expression profiles of pro‐inflammatory cytokines that were indistinguishable from those of wild‐type littermates. This temporal dissociation between vascular maturation and inflammatory onset indicates that neuroimmune activation occurs secondary to the establishment of a cerebrovascular network (Figure S3Z, Supporting Information).

**Figure 3:**
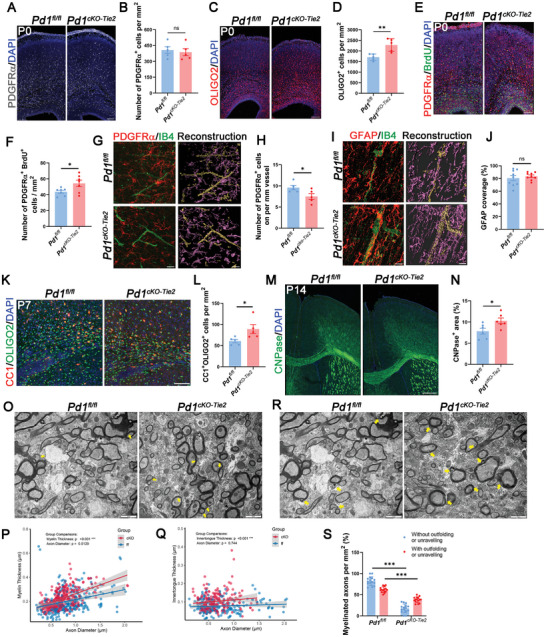
Loss of endothelial PD‐1 promotes OPC differentiation. A) Confocal immunofluorescence images of PDGFRα^+^ cells in *Pd1^fl/fl^* and *Pd1^cKO‐Tie2^* mice cortex at P0. Scale bar, 100 µm. B) Quantification of PDGFRα^+^ cells showed *Pd1^fl/fl^* and *Pd1^cKO‐Tie2^* mice cortex have similar number of OPCs. *n* = 5 mice for each group, two‐tailed unpaired t test. C) Confocal images of OLIGO2^+^ cells in *Pd1^fl/fl^* and *Pd1^cKO‐Tie2^* mice cortex at P0. Scale bars, 100 µm. D) Graph showed the number of OLIGO2^+^ cells per mm^2^ in *Pd1^cKO‐Tie2^* mice cortex was decreased. *n* = 5 mice for each group, p = 0.0052, two‐tailed unpaired t test. E) Brain slices of *Pd1^fl/fl^* and *Pd1^cKO‐Tie2^* mice at P0 were stained with anti‐PDGFRα and BrdU. Scale bar, 100 µm. F) The number of PDGFRα^+^, BrdU^+^ cells in cerebral cortex of *Pd1^cKO‐Tie2^* mice was increased. *n* = 7 mice for each group, p = 0.0437, two‐tailed unpaired t test with Welch's correction. G) Physical contact between PDGFRα^+^ OPCs and IB4^+^ vessels of *Pd1* ^cKO‐Tie2^ mice were increased. Right, 3D reconstruction image. Scale bar, 20 µm. H) Graph showed the number of PDGFRα^+^ cells on IB4^+^ vessels per mm^2^ of *Pd1^fl/fl^* and *Pd1^cKO‐Tie2^* mice. *n* = 5 mice for each group, p = 0.0434, two‐tailed unpaired t test. I) Images showed the physical contacts between GFAP ^+^ astrocytes and IB4^+^ vessels. Right, 3D reconstruction image. Scale bar, 20 µm. J) Graph showed the GFAP coverage (%) on vessel. *n* = 9 mice for each group, two‐tailed unpaired t test with Welch's correction. K) The number of CC1^+^ OLIGO2^+^ cells was increased in the corpus callosum of *Pd1^cKO‐Tie2^* mice at P7. Scale bar, 50 µm. L) Quantification of the CC1^+^ OLIGO2^+^ cells. *n* = 5 mice for each group, p = 0.0371, two‐tailed unpaired t test. M) Immunofluorescent images of CNPase in *Pd1^fl/fl^* and *Pd1^cKO‐Tie2^* mice cortex. Scale bar, 200 µm. N) The CNPase positive area in *Pd1^cKO‐Tie2^* mice cortex was increased. *n* = 6 mice for each group, p = 0.0311, two‐tailed unpaired t test. O) Electron microscopic examination of the myelin from *Pd1^fl/fl^* and *Pd1^cKO‐Tie2^* mice corpus callosum (yellow arrow showed inner tongue). Scale bar, 2 µm. P) Scatterplots showed the myelin thickness versus axon diameter. *n* = 150 axons for each group, p = 0.000711, Wilcoxon test. Q) Scatterplots showed the inner tongue thickness versus axon diameter. *n* = 100 axons for each group, *p* < 0.0001,Wilcoxon test. R) Images of myelin abnormalities in *Pd1^fl/fl^* and *Pd1^cKO‐Tie2^* mice (yellow arrow showed outfoldings or unravelling myelin). Scale bar, 2 µm. S) Proportion of axons with and without outfoldings and unravelling myelin per mm^2^. *n* = 15 images for each group, *p* < 0.0001 (with outfoldings and unravelling), *p* < 0.0001 (without outfoldings and unravelling), multiple t tests, statistical significance determined using the Holm‐Sidak method, with alpha = 0.05. Data are means and SEM, two‐tailed unpaired t‐test, *p* < 0.05(*), *p* < 0.01(**), *p* < 0.001(***), ns not significant.

Numerous studies have reported a significant association between blood vessels and OPCs. Therefore, we investigated whether endothelial PD‐1 affects oligodendrocyte development. We examined the spatiotemporal relationship between the developing vasculature and OPCs at different stages. The results demonstrated a predominantly perivascular distribution of OPCs, with a significantly enhanced spatial correlation observed at P0 (Figure S4A,B, Supporting Information). We then carried out our study with the immunostaining of PDGFRα and observed that the number of these cells remained unaltered at both P0 and P7 periods (**Figure** **3**A,B; Figure S4C,D, Supporting Information). However, we observed a striking increase in the amount of OLIGO2 positive cells in endothelial PD‐1 knockout mice (Figure 3C,D; Figure S4E,F, Supporting Information). To analyze the proliferative capacity of OPCs, we performed BrdU labeling and found more BrdU‐positive OPCs in *Pd1^cKO‐Tie2^* mice cortex compared than in their control littermates (Figure 3E,F; Figure S4G,H, Supporting Information). Although the proliferative capacity of OPCs in *Pd1^cKO‐Tie2^* mice was enhanced, their overall population density remained unchanged, suggesting that accelerated differentiation toward premyelinating oligodendrocytes compensated for the increased progenitor expansion. In addition, we discovered that accelerated OPC differentiation in *Pd1^cKO‐Tie2^* mice correlated with disrupted physical interactions between OPCs and the cerebrovasculature (Figure 3G,H). Notably, this reduction was not affected by astrocyte endfoot coverage (Figure 3I,J). This oligovascular decoupling suggests that endothelial PD‐1 may participate in stabilizes the niche‐anchoring mechanism required for coordinated oligodendrogenesis. Premature oligodendrocyte marker CC1 staining also showed more premature oligodendrocytes within the corpus callosum of *Pd1^cKO‐Tie2^* mice (Figure 3K,L). Comparable outcomes were evident in myelin basic protein (MBP) staining, suggesting that the deletion of endothelial *Pd1* promoted OPC differentiation (Figure S4VI,J, Supporting Information). The enzyme 2'3'‐Cyclic Nucleotide 3'‐Phosphodiesterase (CNPase), primarily found in myelinating oligodendrocytes and crucial for maintaining myelin integrity, exhibited more pronounced signals in the brains of *Pd1^cKO‐Tie2^* mice than in the brains of their wild‐type littermates (Figure 3M,N). Electron microscopy revealed that the myelin sheaths of the adult knockout mice were significantly thicker. But at the same time, a higher proportion of myelin sheaths exhibited inner tongue, outfolding, and unraveling (Figure 3O–S). These findings indicate that the absence of PD‐1 in endothelial cells leads to excessive OPC differentiation and compromised structural integrity. In summary, our findings highlighted the role of endothelial PD‐1 in the modulation of postnatal oligodendrocyte differentiation.

### 2.4 The Absence of Endothelial PD‐1 Regulates the OPCs Differentiation by Secreting GREM1

**Figure 4:**
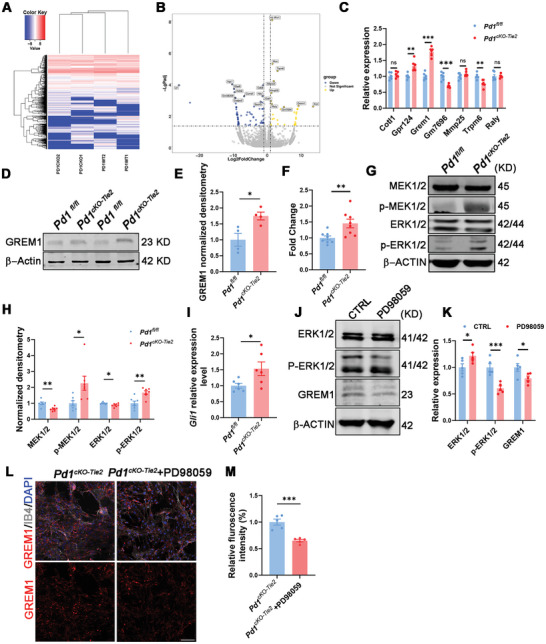
Endothelial PD‐1 regulates GREM1 expression through MEK1/2‐ERK1/2‐GIL1 pathway. A) Heatmap showed global genes were sorted by fold change. B) Volcano plots illustrated the downregulated (blue) and upregulated (yellow) genes in the ECs isolated from *Pd1^fl/f^* ^l^ and *Pd1^cKO‐Tie2^* mice. C) Seven significant upregulated genes were analyzed by RT‐qPCR in purified ECs of *Pd1^fl/fl^* and *Pd1^cKO‐Tie2^* mice, of which *Crem1* has the highest expression. *n* = 6 mice, p = 0.001833 (Gpr124), *p* < 0.0001 (Grem1), p = 0.000199 (Gm7696), p = 0.003015 (Trpm6), multiple t tests, statistical significance determined using the Holm‐Sidak method, with alpha = 0.05. D) Western blot analysis of GREM1 expression levels in ECs from *Pd1^fl/fl^* and *Pd1^cKO‐Tie2^* mice, β‐ACTIN detected as loading control. E) Statistics showed the increased expression of GREM1 in *Pd1^cKO‐Tie2^* mice endothelial cells. *n* = 4 mice for each group, p = 0.0195, two‐tailed unpaired t test. F) GREM1 secretion from the primary cultured ECs was measured by an ELISA kit. *n* = 8 independent experiments, p = 0.0091, two‐tailed unpaired t test. G) Western blot analysis of p‐MEK1/2, MEK1/2, p‐ERK1/2 and ERK1/2 expression levels in endothelia cells isolated from *Pd1^fl/fl^* and *Pd1^cKO‐Tie2^* mice, β‐ACTIN detected as loading control. H) p‐MEK1/2, MEK1/2, p‐ERK1/2 and ERK1/2 normalized densitometry in western blot analysis. *n* = 7 mice for each group, p = 0.001957 (MEK1/2), p = 0.019488 (p‐MEK1/2), p = 0.022378 (ERK1/2), p = 0.002481 (p‐ERK1/2), multiple t tests, statistical significance determined using the Holm‐Sidak method, with alpha = 0.05. I) Quantitative RT‐qPCR analysis of relative *Gli1* mRNA level in the endothelial cell form *Pd1^fl/fl^* and *Pd1^cKO‐Tie2^* brains. *n* = 6 mice for each group, p = 0.0391, two‐tailed unpaired t test. J) Western blot analysis of p‐ERK1/2, ERK1/2 and GREM1 protein levels in HEK293FT cells treated with PD98059, β‐ACTIN detected as loading control. K) p‐ERK1/2, ERK1/2 and GREM1 normalized densitometry in western blot analysis. *n* = 5 individual experiments for each group, p = 0.022415 (ERK1/2), p = 0.000191 (p‐ERK1/2), p = 0.023917 (GREM1). multiple t tests, statistical significance determined using the Holm‐Sidak method, with alpha = 0.05. L) Endothelial cells from *Pd1^cKO‐Tie2^* mice treated with PD98059 could rescue GREM1 overexpression. Scale bar, 100 µm. M) Graph showed the GREM1 normalized fluorescence intensity in endothelial cells. *n* = 5 mice and p = 0.0004, two‐tailed unpaired t test. Data are means and SEM, *p* < 0.05(*), *p* < 0.01(**), *p* < 0.001(***), ns not significant.

To investigate the molecular mechanisms underlying the phenotype caused by the ablation of PD‐1 in endothelial cells, we conducted RNA sequencing on vascular endothelial cells that had been purified using flow cytometry. Based on the sequencing results, we analyzed the transcriptome differences between *Pd1 ^fl/fl^* and *Pd1 ^cKO‐Tie2^* mouse endothelial cells and found that the expression profile of knockout *Pd1* endothelial cells exhibited marked divergence from that of the controls. Differential expression analysis revealed that a substantial number of differentially expressed genes were implicated in essential biological processes, including cell signaling, migration, and angiogenesis (**Figure** **4**A,B).

To investigate the effect of PD‐1 loss on the molecular expression profiles of endothelial cells, we first validated the expression levels of the top seven genes with the most substantial changes using RT‐qPCR. The results revealed a notable upregulation of the BMP antagonist protein GREM1, which was consistent with the RNA‐seq data (Figure 4C). Western blot analysis confirmed that the absence of *Pd1* increased GREM1 protein levels (Figure 4E). ELISA results also showed that GREM1 secretion by the vascular endothelial cells of knockout mice was higher than that of the control group (Figure 4F). Therefore, we hypothesized that GREM1 is a potential downstream target of endothelial PD‐1 loss.

PD‐1 is involved in the regulation of the MEK1/2‐ERK1/2 pathway, while the expression of GREM1 is modulated by the sonic hedghog (Shh)‐GLI family zinc 1 (GLI1) pathway, which interacts with the MEK1/2‐ERK1/2 pathway.^[^ ^38^, ^39^ ^]^ We conducted western blot analyses to assess the protein levels of MEK1/2, p‐MEK1/2, ERK1/2, and p‐ERK1/2. These results suggest that knockout of endothelial PD‐1 promotes MEK1/2‐ERK1/2 signal transduction (Figure 4G,H). RT‐qPCR analysis also indicated that GLI1 expression was increased (Figure 4I). These results indicated that PD‐1 ablation enhanced MEK1/2‐ERK1/2‐GLI1 signaling in endothelial cells. We then used PD98059, an MEK1/2 inhibitor, to investigate whether it could rescue GREM1 expression. The results showed that PD98059 indeed helped block MEK1/2‐ERK1/2 signaling and downregulated GREM1 expression (Figure 4J,K). By applying PD98059, the expression of endothelial GREM1 was rescued, thereby proving that GREM1 expression was regulated by the MEK1/2‐ERK1/2 ‐GLI1pathway (Figure 4L,M).

### 2.5 GREM1 Regulates the OPC Differentiation through BMP/SMAD1/5/SMAD4 Pathway

However, the mechanism through which GREM1 influences the fate of OPCs remains unclear. Prior investigations have established that inhibition of the BMP signaling pathway in OPCs facilitates their differentiation.^[^ ^40^ ^]^ Given that GREM1 functions as a BMP antagonist, we hypothesized that GREM1 affects OPC differentiation by suppressing the BMP signaling cascade.

**Figure 5:**
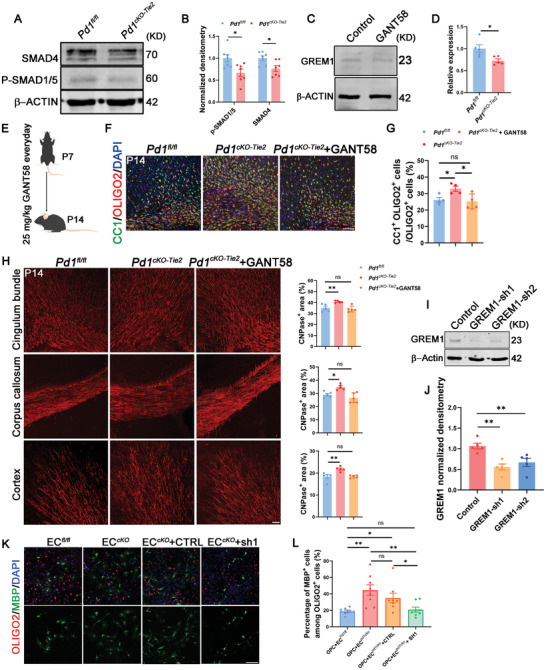
Endothelial GREM1 regulates OPC differentiation through BMP signal pathway. A) Western blot analysis of SMAD4 and p‐SMAD1/5 protein levels in OPCs cocultured with endothelial cells from *Pd1^fl/fl^* and *Pd1^cKO‐Tie2^* mice, β‐ACTIN detected as loading control. B) SMAD4 and p‐SMAD1/5 normalized densitometry in western blot. *n* = 7 mice, p = 0.015088 (p‐SMAD1/5), p = 0.016645 (SMAD4). multiple t tests, statistical significance determined using the Holm‐Sidak method, with alpha = 0.05. C) Western blot analysis of GREM1 protein level in HEK293FT cells treated with GANT58, β‐ACTIN detected as loading control. D) GREM1 expression was decreased when treated with GANT58. *n* = 5 individual experiments for each group, p = 0.0238, two‐tailed unpaired t test. E) The model of GANT58 treatment in *Pd1^fl/fl^* and *Pd1^cKO‐Tie2^* mice. F) The number of CC1^+^ OLIGO2^+^ cell was rescued in *Pd1^cKO‐Tie2^* mice treated with GANT58. Scale bar, 50 µm. G) Percentage of CC1^+^ OLIGO2^+^ cells among OLIGO2^+^ cells. *n* = 4 mice each group, p = 0.0496 (*Pd1^fl/fl^*), p = 0.0414 (*Pd1^cKO‐Tie2^* +GANT58), one‐way ANOVA with Holm‐Sidak's multiple comparisons test. H) The CNPase^+^ expression was rescued in *Pd1^cKO‐Tie2^* mice treated with GANT58. Scale bar, 50 µm. *n* = 5 mice for each group. p = 0.0078 (Cingulum bundle), p = 0.0134 (Corpus callosum), p = 0.0032 (Cortex), one‐way ANOVA with Dunnett's multiple comparisons test. I) Western blot analysis of GREM1 protein levels in ECs treated with GREM1‐shRNA1 or GREM1‐shRNA2. J) Quantification of GREM1 normalized densitometry in western blot. *n* = 5 individual experiments for each group and p = 0.0015 (sh1), p = 0.0085 (sh2), one‐way ANOVA with Dunnett's multiple comparisons test. K) ECs from *Pd1* ^cKO‐Tie2^ mice treated with shRNA successfully rescued OPCs over differentiation. Scale bar, 50 µm. L) The number of MBP^+^ cells were rescued in shRNA group. Scale bar, 50 µm, *n* = 8 individual experiments for each group, p = 0.0057 (*Pd1^cKO‐Tie2^* group compare to *Pd1^fl/fl^ group*), p = 0.0271 (CTRL group compare to *Pd1^fl/fl^* group), p = 0.0478 (CTRL group compare to SH1 group), p = 0.0057 (*Pd1^cKO‐Tie2^* group compare to SH1 *group*), two‐tailed unpaired t test (Welch's t test when SD was not equal). Data are means and SEM, *p* < 0.05(*), *p* < 0.01(**), *p* < 0.001(***), ns not significant.

To test this hypothesis, cortical OPCs were isolated and maintained in vitro, followed by co‐culturing with PD‐1‐deficient endothelial cells to assess the expression levels of p‐SMAD1/5 and SMAD4, which are key indicators of BMP pathway activity. Western blotting revealed a decreased expression of p‐SMAD1/5 and SMAD4 (**Figure** **5**A,B). GANT58 is a GLI1 antagonist that may suppress GREM1 expression.^[^ ^41^ ^]^ Western blotting results showed that GANT58 effectively inhibited the expression of GREM1, further confirming that GREM1 is a downstream factor of GLI1 (Figure 5C,D). We administered 25 mg kg^−1^ GANT58 intraperitoneally to P7 mice daily for seven consecutive days. We found that the inhibition of GLI1 effectively rescued the number of CC1 positive immature oligodendrocytes (Figure 5E–G). Moreover, myelination in the cerebral cortex of the knockout mice was rescued (Figure 5H). We performed *an* in vitro analysis. To specifically silence GREM1, we constructed a GREM1‐specific short hairpin RNA (shRNA) and validated its efficacy using western blot analysis (Figure 5I,J). The results showed that GREM1‐shRNA1 exhibited superior knockdown efficiency. Subsequently, we transfected GREM1‐shRNA1 into *Pd1^cKO‐Tie2^* mouse endothelial cells and co‐cultured them with OPCs. This intervention effectively rescued the overdifferentiation of OPCs (Figure 5K,L). In summary, endothelial cell PD‐1 knockout upregulated MEK1/2‐ERK1/2‐GLI1 signaling, leading to increased expression of the BMP antagonist protein GREM1, whereas the secreted GREM1 protein promoted the differentiation of OPCs into oligodendrocytes by antagonizing the BMP signaling pathway.

### 2.6 Endothelial Pd1 Knockout Mice Show Abnormal Behavior

**Figure 6:**
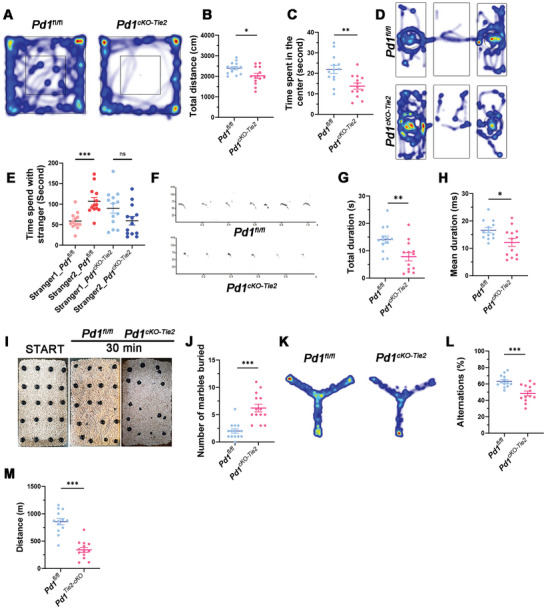
Endothelial PD‐1 deletion induces mice abnormal behavior. A) Representative images of the open field test. B) The total distance of open‐field test was less in *Pd1^cKO‐Tie2^* mice. *n* = 13 mice for each group and p = 0.0168, two‐tailed unpaired t test. C)The time spent in the center was less in *Pd1^cKO‐Tie2^* mice. *n* = 13 mice for each group and p = 0.0035, two‐tailed unpaired t test. D) Representative images of the three chambers social test. E) *Pd1^cKO‐Tie2^* mice spend less time to social with stranger 2 mouse. *n* = 13 mice for each group and p = 0.0002 (*Pd1^fl/fl^* group) and ns (*Pd1^cKO‐Tie2^* group), two‐tailed unpaired t test. F) Representative USV spectrograms in isolated P7 pups. G) The total duration of calls was decreased in *Pd1^cKO‐Tie2^* pups. *n* = 13 pups for each group and p = 0.005, two‐tailed unpaired t test. H) the mean duration of calls was decreased in *Pd1^cKO‐Tie2^* pups. *n* = 13 pups for each group and p = 0.0174, two‐tailed unpaired t test. I) Representative images of marble buried test. J) The number of marbles buried in *Pd1^cKO‐Tie2^* mice group was increased. *n* = 14 mice for each group and *p* < 0.0001, two‐tailed unpaired t test. K) Representative images of the alternative Y maze. L) The percentage of alternation of *Pd1^cKO‐Tie2^* mice was decreased. *n* = 13 mice for each group and p = 0.0003, two‐tailed unpaired t test. M) Graph showed the total distance in treadmill test. *n* = 13 mice for each group and *p* < 0.0001, two‐tailed unpaired t test. Data are means and SEM, *p* < 0.05(*), *p* < 0.01(**), *p* < 0.001(***), ns not significant.

Since endothelial PD‐1 deletion induces abnormal cerebrovascular development, we hypothesized that these abnormalities might influence mouse behavior. To validate this hypothesis, an open‐field test was conducted using *Pd1^cKO‐Tie2^* and *Pd1^fl/fl^* mice. The *Pd1^cKO‐Tie2^* mice spent less time in the center of the arena and exhibited a reduced total distance traveled, suggesting that the exploratory behavior and locomotor activity of *Pd1^cKO‐Tie2^* mice were impaired (**Figure** **6**A–C). To determine whether *Pd1^cKO‐Tie2^* mice exhibit abnormal social abilities, three‐chamber social interaction tests were used. *Pd1^cKO‐Tie2^* mice show no specific preference for strangers 1 or 2. These results indicated that *Pd1^cKO‐Tie2^* mice had abnormal social abilities (Figure 6D,F). In addition, an ultrasonic vocalization (USV) test showed that both the total call duration and mean call duration of the *Pd1^cKO‐Tie2^* pups at P7 were shorter than those of the wild‐type pups when isolated from their mothers and littermates (Figure 6G–I). These findings collectively suggested that the deletion of PD‐1 in endothelial cells leads to significant behavioral and social deficits in mice. Subsequently, a marble barrier test was performed, and we observed that *Pd1^cKO‐Tie2^* mice buried a greater number of marbles than *Pd1^fl/fl^* mice, indicating that endothelial ablation of PD‐1 mice exhibited stereotypical behavior (Figure 6J,K). A novel thing recognition test was used to evaluate the recognition memory. The results revealed that the endothelial *Pd1* knockout did not affect mouse recognition memory (Figure S5A,B, Supporting Information). We then assessed short‐term working memory and found that PD‐1 knockout mice had worse short‐term working memory (Figure 6L,M). To evaluate anxiety levels, we conducted an elevated plus maze test and found no significant differences between the two groups (Figure S5C–E, Supporting Information). Subsequently, a forced swim test was performed, and the data illustrated that the loss of endothelial PD‐1 did not induce depression‐like states (Figure S5F, Supporting Information). Because myelination is related to motor ability, we evaluated the motor ability of *Pd1^cKO‐Tie2^* mice. Only physical endurance, not grip force or motor balance, was affected (Figure 6N; Figure S5G,H, Supporting Information). Collectively, the results of these behavioral tests indicated that *Pd1^cko‐Tie2^* mice exhibited abnormal behavior and that their motor abilities were slightly affected.

## 3 Discussion

Proper coordination of angiogenesis and oligovascular coupling is essential for maintaining normal brain function during development. Our study reveals a new mechanism highlighting the significance of brain endothelial PD‐1 in regulating the OPC response during this process. The absence of endothelial PD‐1 impairs angiogenesis and the BBB by inhibiting the canonical Wnt/β‐ catenin signaling pathway and disrupts oligodendrogenesis through the crosstalk between ERK and BMP pathways. In summary, our study highlights the vascular system of the brain as a critical niche that supports OPCs during important periods of cortical development.

The canonical Wnt/β‐catenin signaling pathway plays a crucial role in promoting angiogenesis by stimulating the proliferation and migration of endothelial cells, which is essential for the formation of new blood vessels.^[^ ^42^, ^43^ ^]^ Our study reveals a paradoxical phenomenon that endothelial PD‐1 knockout disrupts Wnt/β‐catenin signaling, driving a vascular phenotype of hyperangiogenesis alongside BBB immaturity. This dual outcome underlines the multifaceted role of Wnt signaling in cerebrovascular regulation. The establishment of a functional vasculature requires endothelial cells to enter a quiescent state during development; otherwise, pathological overgrowth occurs. This process requires activation of the Wnt/β‐catenin pathway in endothelial cells.^[^ ^44^ ^]^ Previous research using *Fzd4* ^ECKO^ and *Lrp5* ^−/‐^ models have demonstrated considerable increases in endothelial cell proliferation, cyst formation, and vessel dilation.^[^ ^45^, ^46^ ^]^ Our findings were consistent with this theory, that inhibiting Wnt/β‐catenin leads to pathological overgrowth of blood vessels. In addition to its role in brain angiogenesis, Wnt/β‐catenin signaling in endothelial cells is important for the development and maintenance of the BBB, as shown by the loss of barrier integrity following a sudden decrease in Wnt signaling and its recovery after reactivation.^[^ ^46^, ^47^ ^]^ Activation of Wnt signaling is marked by increased protein expression of several Wnt ligands, such as Wnt‐3a and Wnt‐5a, as well as β‐catenin, and the pathway is inhibited by GSK‐3β, which promotes the degradation of β‐catenin.^[^ ^48^ ^]^ Here we observed that that ablation of endothelial PD‐1 disrupted the Wnt/β‐catenin signaling, leading to tight junction impairment and BBB immaturity. Previous studies have found that PD‐1 in neural stem cells can regulate Wnt downstream molecules by modulating the activation of the AKT/GSK‐3β/β‐catenin.^[^ ^29^ ^]^ However, the specific mechanism by which endothelial cell PD‐1 regulates the Wnt/β‐catenin signaling pathway remains unknown.

The existence of hypervascularization may also indicate compensatory pathway activation overriding Wnt's angiogenic effects, potentially leading to abnormal vessel generation when Wnt/β‐catenin signaling is inhibited, such as VEGF pathway, TGF‐β/BMP pathway, and Shh pathway.^[^ ^49^, ^50^, ^51^ ^]^ Our RNAseq data demonstrated a significant increase in the expression of genes such as *Mmp25*, *Gpr124*, and *Grem1*. These genes are involved in angiogenesis via different signaling pathways. The simultaneous upregulation of these genes suggests the presence of complex interactions between different angiogenic pathways during brain development. Strikingly, excessive vasculature is a hallmark of nonproductive angiogenesis (NPA), characterized by excessive branching and perfusion deficits.^[^ ^52^, ^53^ ^]^ Our study emphasized the delicate balance between angiogenesis and vascular stability during the development of cerebrovascular disease. Although the Wnt/β‐catenin signaling pathway plays a crucial role in angiogenesis during development, its inhibition may also result in pathological angiogenesis. Future research should focus on the spatial and post‐translational dynamics. Clinically, these findings suggest caution against broad PD‐1 inhibition in neurovascular contexts and point to therapeutic opportunities for diseases such as cerebral amyloid angiopathy. Future studies should explore methods to promote angiogenesis while maintaining vascular stability and functionality.

The BMP pathway significantly affects OPC differentiation by inhibiting its maturation into oligodendrocytes, and the elimination of BMP signaling can lead to enhanced remyelination.^[^ ^40^ ^]^ GREM1, a BMP antagonist, is crucial for embryonic development, including the normal growth and patterning of limbs and organs, such as the kidneys and skeleton.^[^ ^54^, ^55^, ^56^ ^]^ Our study identified a novel regulatory axis wherein endothelial PD‐1 deletion activates the MEK1/2‐ERK1/2‐GLI1 signaling cascade, driving GREM1 overexpression. Elevated GREM1 secretion inhibits BMP‐SMAD1/5‐SMAD4 signaling in OPCs, disrupting quiescence maintenance. This pathological suppression forces premature OPC differentiation into mature oligodendrocytes, bypassing the precise developmental timing required for controlled myelination. While myelination enables saltatory conduction, which is essential for neural network efficiency,^[^ ^57^ ^]^ accelerated oligodendrogenesis carries significant risks. Untimely differentiation depletes the OPC pool necessary for lifelong myelin plasticity,^[^ ^58^ ^]^ mirroring observations in autism spectrum disorder models, where disrupted OPC kinetics impair cortical circuit refinement.^[^ ^59^ ^]^ Furthermore, an abnormal myelin thickness distribution alters action potential synchronization across neural ensembles,^[^ ^60^ ^]^ potentially explaining the cognitive inflexibility observed in our behavioral assays.

During early embryogenesis, the immature BBB provides partial insulation from systemic influences, while embryonic microglia exist in a neurotrophic phagocytic state that tolerates local perturbations.^[^ ^61^, ^62^ ^]^ This protective milieu diminishes postnatally as BBB maturation completes. However, a compromised BBB allows the infiltration of peripheral immune cells and plasma proteins into the CNS, which can activate microglia and initiate inflammatory responses.^[^ ^63^ ^]^ Elevated inflammatory cytokines such as IL‐1β, IL‐6, and TNF‐α can further stimulate microglia, creating a pro‐inflammatory environment.^[^ ^52^ ^]^ We observed that deletion of endothelial PD‐1 led to microglial activation and myelination deficits. This dual‐hit mechanism, involving both intrinsic dysregulation of OPCs and extrinsic neuroinflammatory processes, could be the underlying cause of neurodevelopmental disorders characterized by myelination deficits. The clinical similarities are remarkable, with patients with schizophrenia exhibiting both microglial activation and aberrant frontostriatal myelin, whereas genetic studies have implicated BMP pathway dysregulation in autism spectrum disorder susceptibility.^[^ ^64^, ^65^ ^]^ Our findings position endothelial PD‐1 as a critical integrator of vascular and gliogenic signaling, where its loss disrupts the coordination between angiogenesis and myelination programs. However, the precise mechanisms that coordinate the endothelial cells, OPCs, and microglia remain unclear. Future studies employing single‐cell transcriptomics could delineate the spatiotemporal crosstalk between PD‐1‐deficient endothelia, OPCs, and microglia, whereas longitudinal diffusion tensor imaging may reveal how premature myelination trajectories contribute to circuit‐level dysfunctions.

Our data revealed a cell type‐specific vulnerability to endothelial PD‐1 ablation. Although OPC differentiation was profoundly accelerated by GREM1 mediated BMP suppression, cortical neurogenesis remained intact. This phenomenon can be attributed to several interconnected mechanisms. The exquisite sensitivity of OPCs to BMP signaling threshold is unlike neuronal progenitors that primarily rely on Notch for fate determination,^[^ ^66^ ^]^ OPCs require BMP positioning to balance self‐renewal and differentiation.^[^ ^40^ ^]^ The GREM1 upregulation in PD‐1 knockout endothelial may drive local BMP activity below the critical level required for OPC maintenance, whereas neuronal progenitors in adjacent niches receive compensatory BMP signals from Noggin.^[^ ^67^, ^68^ ^]^ In addition, the spatiotemporal decoupling of neurovascular interactions coincides with peak myelination windows, whereas neurogenic vascular guidance occurs earlier, a period when endothelial PD‐1 expression is minimal.^[^ ^69^ ^]^ Endothelial PD‐1 is considered a spatiotemporal safeguard within the neurovascular niche that orchestrates gliovascular coupling during postnatal myelination without overriding embryonic neurogenic programs. However, the precise mechanisms underlying this phenomenon need to be further explored. Future studies using inducible knockout models could test whether PD‐1 maintains similar niche functions in adult remyelination, with implications for demyelinating disorders, such as multiple sclerosis, where BMP antagonists show therapeutic promise.

## 4 Limitations

The main limitation of our study is the use of Tie2‐Cre mice for endothelial cell‐specific gene deletion. Although Tie2‐Cre mice are widely used because of their robust expression of Cre recombinase in endothelial cells, Tie2 is also expressed Cre recombinase in hematopoietic cells and certain myeloid lineages.^[^ ^53^, ^70^ ^]^ This nonspecific expression may have led to confounding results. The expression of Cre recombinase in hematopoietic cells may influence immune cell function and contribute to the changes in microglial activation and neuroinflammation observed in our study. This overlap makes it difficult to determine whether phenotypic outcomes are solely driven by endothelial‐specific PD‐1 deletion or are partially influenced by alterations in hematopoietic cells.

To overcome this limitation, future studies should use more specific brain endothelial Cre driver lines to minimize recombination in non‐endothelial cells. For instance, SP‐A‐cre mice, which use the surfactant protein A (SP‐A) promoter to drive Cre expression specifically in cerebrovascular endothelial cells, would provide enhanced specificity.^[^ ^71^ ^]^ Additionally, Tie2‐Dre; Mfsd2a‐crexE mice are a double‐recombination approach that allows for gene deletion in specific endothelial subpopulations (such as choroid plexus endothelial cells), offering a more refined targeting strategy.^[^ ^57^ ^]^ These Cre lines would significantly improve the precision of endothelial‐specific gene manipulation and reduce off‐target effects, thereby enhancing the accuracy of attributing the observed phenotypes to endothelial gene deletion. Furthermore, the use of inducible Cre systems such as the tamoxifen‐inducible CreERT2 line could provide rigid temporal control over gene modulation, enabling the investigation of gene function during a specific period. Combining such strategies with lineage‐tracing experiments and conditional knockout models will enhance our understanding of the role of endothelial PD‐1 in cerebrovascular diseases and brain development.

## 5 Conclusion

In summary, our study highlighted the critical role of endothelial PD‐1 in brain development, particularly in angiogenesis, BBB formation, and oligodendrocyte development. Endothelial PD‐1 knockout impairs angiogenesis and BBB integrity by inhibiting Wnt/β‐catenin signaling, leading to pathological overgrowth of blood vessels and BBB immaturity. Additionally, it disrupts oligodendrogenesis through crosstalk between the ERK and BMP pathways, resulting in premature oligodendrocyte differentiation. Our research offers novel insights into the regulatory relationship between the cerebrovasculature and OPCs. Future research should focus on the spatiotemporal dynamics of PD‐1 in vascular and gliogenic signaling and explore therapeutic opportunities for diseases such as cerebral amyloid angiopathy and multiple sclerosis.

## 6 Experimental Section

### Animals

Pregnant ICR and C57BL/6J mice (aged 8–10 weeks) were obtained from Vital River Laboratories (Beijing, China). The *Pd1^flox/flox^* mice were obtained from the Laboratory Animal Center of the Institute of Zoology, Chinese Academy of Sciences. *Pd1^flox/flox^* mice were mated with Tie2‐Cre transgenic mice (Shanghai Model Organism Center) to obtain endothelial‐specific *Pd1* knockout mice, *Pd1^cKO‐Tie2^* mice. Mice were raised and kept in an environment with a temperature of 22–25 °C and a 12 h light‐dark cycle while being supplied with sufficient food and water. All animal experiments were performed in accordance with guidelines approved by the Animal Experiment Ethics Review Committee and Experimental Animals Management Regulations of the Institute of Zoology, Chinese Academy of Sciences (IOZ‐IACUC‐2024‐143). Tail genomic DNA PCR was used to genotype the experimental animals. The experimental procedures and primer sequences were consistent with those provided by the supplier.

### Cell Culture

Human embryonic kidney 293T cells (HEK293FT) were grown in high‐glucose Dulbecco's modified Eagle's medium (DMEM) supplemented with 10% fetal bovine serum and 1% penicillin and streptomycin. The medium was replaced every 72 h.

### Primary Culture of Mouse Brain Endothelial Cells

The cerebral cortices of embryonic or postnatal mice were harvested in ice‐cold PBS and fine minced before Papain digestion for 5 min at 37 °C. The Papain was removed and rinse the tissue with DMEM, followed by repeated blowing of the tissue with pipettes. Cells were harvested by centrifugation at 128 × g for 5 min, and the resulting cell suspension was filtered through a 70 µm nylon cell mesh to remove tissue clumps. The filtered cells were resuspended in red blood cell lysis buffer for 2 min. Following centrifugation at 128 × g for 5 min, the collected cells were resuspended in 2% FBS‐PBS and labeled with anti‐CD31‐FITC and anti‐CD45‐PE. Labeled cells were sorted by fluorescence‐activated cell sorting (FACS) using a Calibur cytometer (Becton Dickinson) following the manufacturer's instructions. The sorted cells were incubated in endothelial conditioned medium supplemented with antibiotics and seeded on culture plates pretreated with rat tail collagen type I. The culture medium was replaced every 72 h.

### Primary Mouse OPC/OL Culture

Embryonic or postnatal mice were sacrificed and the cerebral cortices were carefully isolated and placed in ice‐cold PBS. Peeling off the meninges and washing with DMEM/F12, the tissues were finely chopped and followed by Papain digestion for 5 min at 37 °C. After centrifugation at 128 × g for 5 min, suspended cells were filtered through 70 µm filter nylon mesh, and the collected cells were seeded onto 24‐well plates precoated with ploy‐D‐lysine and culture in DMEM/F12 media supplemented with 10% fetal bovine serum, 20 ng mL^−1^ PDGF‐AA, 10 ng mL^−1^ FGF, N‐2, B27and antibiotics. The medium was changed every two days. To induce OPC differentiation, the proliferation medium was replaced with DMEM/F12 containing 10% FBS, T3, CNTF, and antibiotics.

### Immunostaining

Embryonic or postnatal brains were dissected, fixed in 4% paraformaldehyde (PFA), and dehydrated in 30% sucrose for at least 24 h at 4 °C. Dehydrated brains were then mounted in Tissue‐Tek OCT and cut into 15 or 40 um slices. Brain slices or cell samples were fixed with 4% PFA for 30 min, washed thrice with phosphate‐buffered saline supplemented with 0.1% (cells) or 1% (tissues) Triton X‐100 (PBST), and blocked with 5% bovine serum albumin (BSA) for an hour. Samples were incubated overnight with primary antibody at 4 °C. On the second day, the slices were washed three times with PBS containing 0.1% or 1% Triton X‐100 and incubated with secondary antibody for 1 h. Samples were then wash three times with PBST and treated with 4′,6‐diamidino‐2‐phenylindole (DAPI 2 µg µL^−1^) for 4 min. The slices were mounted onto coverslips containing 50% glycerin. Images were captured using a Leica Stellaris confocal microscope and processed and analyzed using ImageJ and Prism 8 software. 3D reconstruction images were generated the Imaris 9.0.1 software.

### Western Blot

RIPA buffer supplemented with 1% PMSF protease inhibitor and 1% cocktail protease inhibitor was used to lyse the collected tissues or cells. Tissues were vulnerable to ultrasonic fragmentation and centrifuged at 13 500 × g at 4 °C for 10 min to collect the supernatant. Protein levels were measured using Pierce BCA Protein Assay Reagent following the manufacturer's instructions. Protein samples were separated using sodium dodecyl sulfate‐polyacrylamide gel electrophoresis after boiling for 10 min. Protein samples were then transferred to nitrocellulose membranes and blocked in 5% skim milk or 5% BSA (dissolved in PBS containing 0.05% Tween‐20) for 1 h on an orbital shaker. This was followed by an overnight incubation with primary antibodies at 4 °C. The following day, the nitrocellulose membranes were washed thrice for 10 min each with TBST (PBS with 0.05% Tween20). Secondary antibodies were then applied to the nitrocellulose membranes. After incubation, the membranes were washed with TBST and visualized using an Odyssey two‐color infrared laser‐scanning imaging system. Images were processed using Odyssey and ImageJ software.

### Preparation of the Plasmid

The pSicoR‐GFP vector was used to integrate the GREM1 shRNA constructs. The sequences of the two GREM1‐targeting shRNAs were as follows: GREM1‐sh1, 5′‐GCAACAGCCGCACTATCATCACTCAGATGATGATAGTGCGGCTGTTGC‐3′ and GREM1‐sh2, 5′‐CGTTGTCGGCGTGATAGTAGTGAGTCTACTACTATCACGCCGACAACG‐3′.

### Elisa Assay

P0 mouse brain endothelial cells were isolated and cultured in Endothelial Conditional Medium at 37 °C. Culture supernatants were harvested for ELISA. Supernatants were assayed using a Mouse Gremlin ELISA Kit.

### Quantitative Real‐Time PCR

RNA was extracted using TRIzol reagent according to the manufacturer's protocol. A FastQuant RT kit was used to synthesize cDNA. Quantitative real‐time PCR was performed using a SuperReal Quantitative Assay Kit on an ABI7500 Real‐Time PCR System (Applied Biosystems). For detailed steps, please refer to the instruction manual.

### BrdU Labeling

Pregnant mice were injected with 50 mg kg^−1^ BrdU 2 h prior to embryonic brain extraction and fixation. For BrdU labeling of cells, they were treated with 10 µg mL^−1^ BrdU 2 h before undergoing immunofluorescence cell staining.

### Detection of Blood Brain Barrier Integrity

Detection was performed as previously described.^[^ ^9^ ^]^ Briefly, 40 uL of Alexa Fluor 555 cadaverine (1 mg mL^−1^) was injected intraperitoneally for postnatal mice (P7). Alexa Fluor 555 Cadaverine was circulated for 2 h. The mice were sacrificed and perfused intracardially with PBS. Brians were then fixed with 4% PFA overnight at 4 °C before dehydration with 30% glucose.

### Detection of Evans Blue Leakage

Eight‐week‐old mice were injected intraperitoneally with 100 µL of 2% Evans blue dye for 24 h. After intracardial perfusion with PBS, the brains were dissected and fixed in 4% PFA.

### Electron Microscopy Analysis

Electron microscopy was performed as previously described.^[^ ^59^ ^]^ The mice were anesthetized and perfused with phosphate buffered saline (PBS), the brains were isolated, and the corpus callosum was extracted. The corpus callosum was fixed with glutaraldehyde/osmium tetroxide for 24 h at 4 °C. The fixed tissues were first treated with 1% OsO_4_ and 1.5% potassium ferricyanide aqueous solution for 2 h. After ultrathin sectioning, the sections were stained with uranyl acetate and lead citrate and examined using a transmission electron microscope.

### GANT58 Treatment

P7 mice were intraperitoneally injected with 25 mg kg^−1^ GANT58 daily for seven consecutive days. On day 8, the mice were sacrificed and their brains were isolated and fixed with 4% PFA.

### Behavioral Studies

P7 pups were used for ultrasonic vocalization and 8–12 weeks old mice for other experiments.

### Open‐Field Test

Mice aged 8–12 weeks were individually placed in a 60 cm × 60 cm × 60 cm open‐field apparatus. Within 5 min, the movement trajectories of the mice were recorded using the behavior analysis software EthoVision XT14. The mice were returned to the rearing cage after recording, and the box was cleaned with 75% alcohol to prevent odors in future studies.

### Elevated Plus Maze Experiment

The maze had two closed arms (25 cm × 5 cm) and two open arms (25 cm × 5 cm), and was 50 cm off the ground. EthoVison XT14 behavior analysis software was used to place the mouse in the center of the maze and record how long it spent in both the open and closed arms throughout a 5 min period. The researcher remained at least one meter away from the maze throughout the experiment. After the trial was completed, the maze was completely cleaned with 75% alcohol and the mice were returned to the rearing cage.

### Alternating Y‐Maze

The Y‐maze contained three arms of the same length. The mouse was placed in the middle of the Y‐maze, allowed to explore the maze freely for 5 min, and the behavior of the mouse was recorded using the EthoVision behavior analysis software. At the end of the experiment, the mice were returned to the rearing cage, and the Y‐maze was thoroughly cleaned with 75% alcohol.

### Three‐Chamber Social Experiment

This experiment consisted of the following three phases: The first phase was the acclimation phase, which consisted of placing two empty wire cages in the left and right compartments and allowing the mice to acclimate for 5 min. In the second step, after acclimatization, age‐ and sex‐matched strangers1 were assigned to the left wire cage, and the experimental mice were placed in the middle compartment and allowed to explore freely for 5 min. Third, sex‐ and age‐matched stranger mouse 2 was assigned to the right wire cage, and the movement trajectory of the experimental mouse was recorded using EthoVision behavioral analysis software. At the end of the experiment, the box was cleaned using 75% alcohol.

### Forced Swimming Experiment

A cylindrical glass container, 35 cm high and 20 cm in diameter, was filled with water at 25 °C, the mouse was placed in the water and the mouse behavior was recorded for 5 min. At the end of the experiment, the cylindrical glass container was cleaned with 75% alcohol and the water was changed.

### Marble Embedding Experiment

This experiment was performed in a standard rearing cage familiar to the mice. There A 5 cm thick padding in the cage. Twenty black marbles were gently placed on the padding, returned to the cage, and allowed to move freely for 30 min. The buried marbles were counted.

### Analysis of Pup Ultrasound Recordings

An ultrasound recording microphone (Avisoft Bioacoustics, Berlin, Germany) was placed in a soundproof box. It was connected to a computer via an E‐MU0404 USB audio device. Real‐time recording was performed using the Avisoft software, followed by data analysis using the Avisoft SASLab Pro software. At the beginning of the experiment, pups at 7 d after birth were removed from their littermates and mothers and acclimatized under a microphone for 5 min. The recording parameters of the device include a Frequency of 25–140 kHz; Fast Fourier Transform parameters (5512 FFT length, Hamming window, 100% frame, and 75% time window overlapping), and frequency and time resolutions of the spectrum of 488 Hz and 0.512 ms. The call detection parameters were held for 10 ms, the threshold was set to 40 dB, the ultrasound filter was set to 30 kHz, and the noise reduction filter was set to 40 dB. The entire process lasted 5 min. The box was cleaned using 75% alcohol at the end of each session.

### Treadmill Test

A six‐channel treadmill apparatus was used (12 m min^−1^). The mice were acclimated to the treadmill for one day in advance. On the day of the experiment, the running distance of the mice was recorded after they were placed on a treadmill (at a constant speed). The experiment was terminated when the mice were subjected to five consecutive electric shocks within 1 min. At the end of the experiment, the mice were returned to the rearing cage, and the treadmill instrument was thoroughly cleaned with 75% alcohol.

### Rotarod Test

Mice were acclimated to the rotary‐rod device one day prior to testing. The apparatus was set in the acceleration mode from 4 to 40 rpm for 300 s. The rod was initially rotated at a constant speed of 4 rpm to allow all the mice to be positioned in their respective lanes. When all mice are “ready,” the rod will accelerate from 4 to 40 rpm in 300 s. The time taken for each mouse to fall off the bar was recorded. At the end of the experiment, the mice were returned to the rearing cage, and the rotarod instrument was thoroughly cleaned with 75% alcohol. The test was repeated three times and the average was recorded.

### Grip Strength Test

The experimental mice were placed on a grip strength meter and the tail of the mouse was gently pulled to allow it to grasp the probe with its forelimbs. The reading was recorded on a grip strength meter as the experimenter applied maximum force to the experimental mouse. The measurements were repeated three times and the average was recorded.

### RNA‐Sequencing Analysis

RNA was isolated from P0 brain endothelial cells of *Pd1^flox/flox^* and *Pd1^cKO‐Tie2^* mice using TRIzol. RNA was quantified using an Agilent 2100 Bioanalyzer (Annoad Genomics). High‐throughput sequencing was performed using the Illumina HiSeq 2500 platform (Annoad Genomics). Statistical significance was attributed to genes exhibiting a change of at least two‐fold with a p value of 0.05 or less. The sequence data were available in the Gene Expression Omnibus (GEO) of the National Center for Biotechnology Information (NCBI) under accession number GSE267172.

### Quantification and Statistical Analysis

Before conducting the analysis, the data were first subjected to a normal distribution analysis. Statistical analysis was performed using an unpaired two‐tailed Student's t‐test when comparing two groups (if the data did not conform to a normal distribution, the Wilcoxon test or Welch's correction was used) and one‐way ANOVA or multiple t‐tests when comparing more than two groups. The specific statistical methods used for the One‐way ANOVA and multiple tests were provided in the figure legend. GraphPad Prism 8.0 software and R were used for all statistical analyses. Sample sizes (n) were shown in the figure legends. Data were presented as means ± SEM. *p* < 0.05(*), *p* < 0.01(**), and *p* < 0.001(***), ns not significant.

The detailed information of the experimental materials and reagents involved in this study, including name, source, and catalog number, are listed in the Supporting Information.

## Conflict of Interest

The authors declare no conflict of interest.

## Author Contributions

T.H., F.J., and J.J. designed the study. T.H. conducted experiments, analyzed data and interpreted the data. M.Z. and J.Q. provided some important materials and given technical help. Y.W. and S.L. provided technical help and valuable advice. C.D. performed some experiments. T.H. drafted the manuscript based on comments from all the authors. F.J. and J.J. supervised the project and obtained funding support.

## Supporting information



Supporting Information

Supplemental Data 1

## Data Availability

RNA‐seq datasets generated and analyzed in this study were deposited in NCBI Sequence Read Archive (SRA). All sequencing data reported in this paper have been deposited in NCBI's GEO under the accession number GSE267172. The source data of confocal images of this paper have been deposited in Bioimage Archive under the accession number S‐BIAD1416.
