# Supplementary material for: Endothelial PD‐1 Regulates Vascular Homeostasis and Oligodendrogenesis during Brain Development
Source: Adv Sci (Weinh). 2025 Feb 27;12(16):2417410. doi: 10.1002/advs.202417410 (PMC12021089; doi:10.1002/advs.202417410)
Supplement: Supplementary file 1 — Supporting Information [file ADVS-12-2417410-s001.docx]

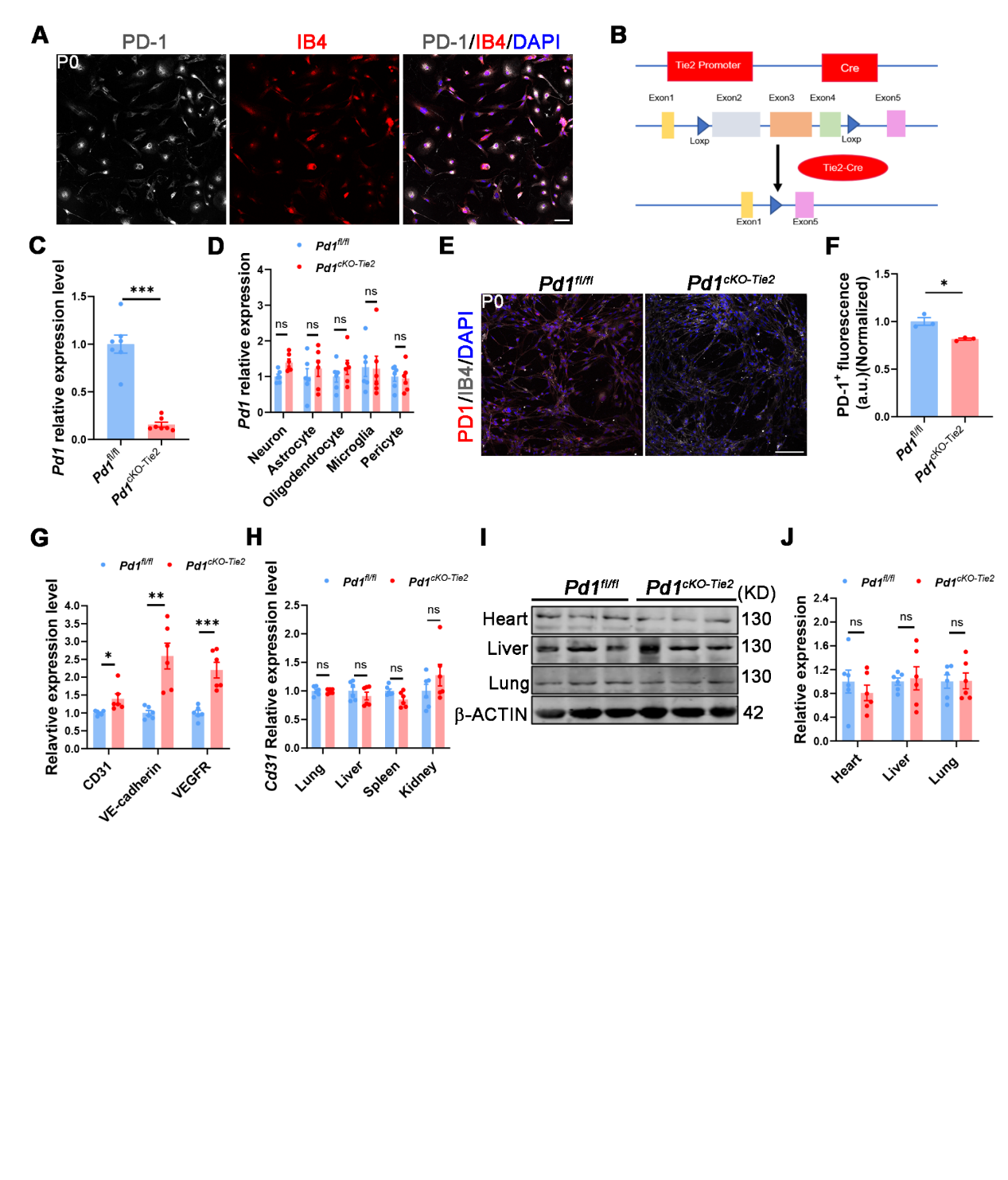


**Figure S1.** Endothelial PD-1 knockout promotes brain vessels development. A) Immunofluorescence staining of PD-1 and IB4 in cultured endothelial cells. Scale bar, 50 μm. B) Schematic construction of endothelial *Pd1* conditional knockout mice. C) RT-qPCR analysis of *Pd1* mRNA level showed that *Pd1^cKO-Tie2^* mice brains endothelial cells *Pd1* expression had a significant decreased. n = 7 mice for each group and p < 0.0001, two-tailed unpaired t test. D) RT-qPCR analysis of relative *Pd1* mRNA level in the *Pd1^fl/fl^* and *Pd1^cKO-Tie2^* mice brains purified neuron, astrocyte, oligodendrocyte, microglia and pericyte. n = 6 mice for each group. multiple t tests, statistical significance determined using the Holm-Sidak method, with alpha = 0.05. E) The expression of PD-1 was decreased in *Pd1^cKO-Tie2^* mice brain endothelial cells. Scale bar, 100 μm. F) Normalized fluorescence intensity of PD-1 from *Pd1^fl/fl^* and *Pd1^cKO-Tie2^* mice brain endothelial cells. n = 3 mice for each group and p = 0.0111, two-tailed unpaired t test. G) RT-qPCR analysis of relative CD31, VE-Cadherin and VEGFR mRNA level in *Pd1^fl/fl^* and *Pd1^cKO-Tie2^* mice brains. n = 6 mice for each group, p = 0.021228 (CD31), p = 0.001468 (VE-cadherin), p = 0.000397 (VEGFR). multiple t tests, statistical significance determined using the Holm-Sidak method, with alpha = 0.05. H) Quantitative RT-qPCR analysis of relative CD31 mRNA level in *Pd1*^fl/fl^ and *Pd1*^cKO-Tie2^ mice lung, liver, spleen and kidney. n = 6 mice for each group. multiple t tests, statistical significance determined using the Holm-Sidak method, with alpha = 0.05. I) CD31 expression in *Pd1^fl/fl^* and *Pd1^cKO-Tie2^* mice heart, lung and liver, β-ACTIN detected as loading control. J) CD31 normalized densitometry in western blot analysis. n = 6 mice for each group. multiple t tests, statistical significance determined using the Holm-Sidak method, with alpha = 0.05. Data are means and SEM, p < 0.05(*), p < 0.01(**), p < 0.001(***), ns not significant.


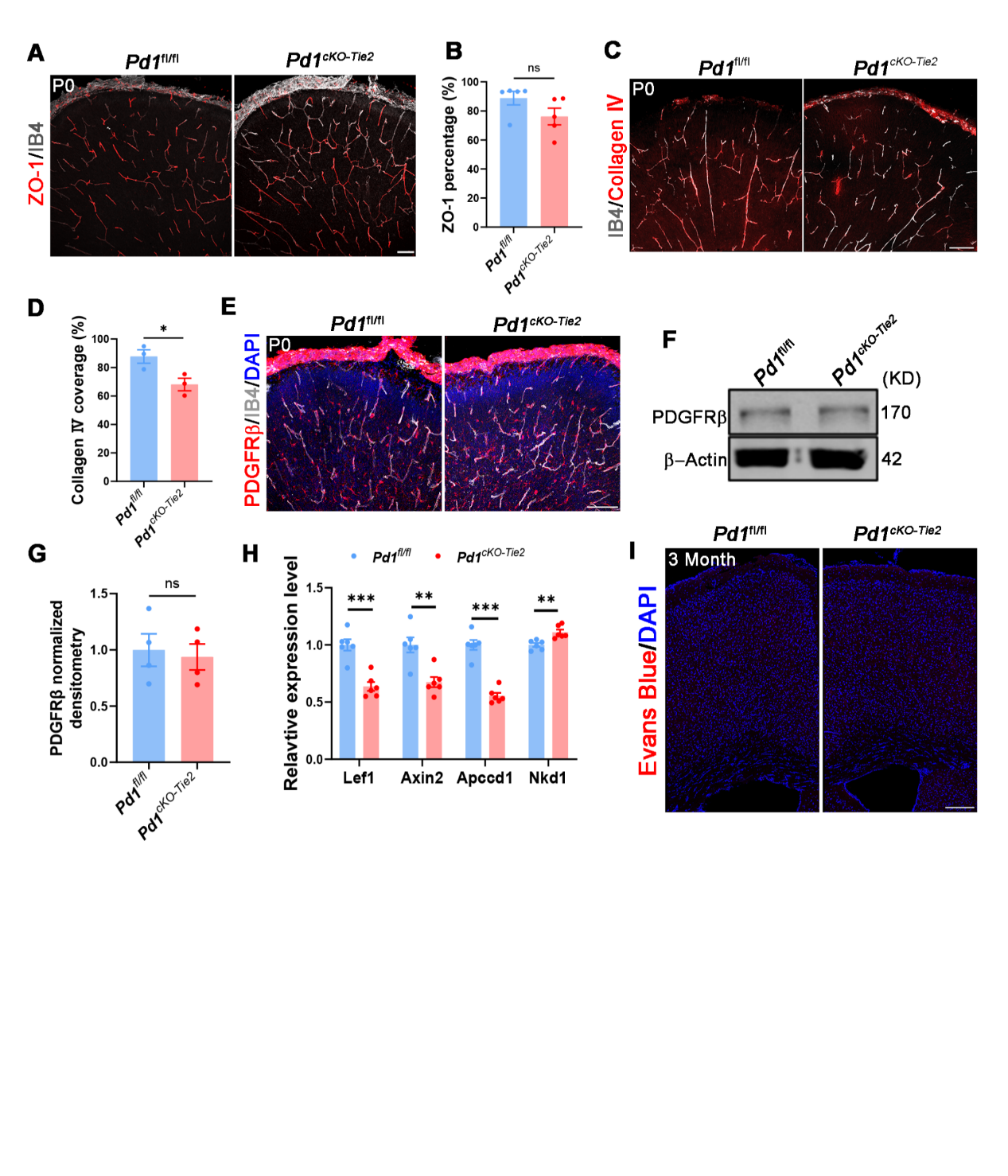


**Figure S2.** Endothelial PD-1 ablation affects blood brain barrier formation in early postnatal period but not adult period. A) The expression of ZO-1 in *Pd1^cKO-Tie2^* mice was similar to *Pd1^fl/fl^* mice. Scale bar, 50 μm. B) Graph showed the percentage of ZO-1 coverage. n = 5 mice for each group. two-tailed unpaired t test. C) Collagen Ⅳ expression was decreased in *Pd1^cKO-Tie2^* cortex at P0. Scale bar, 100 μm. D) Quantification of vessel’s collagen Ⅳ coverage (%). n = 3 mice for each group and p = 0.0385. two-tailed unpaired t test. E) The expression of PDGFRβ in *Pd1*^cKO-Tie2^ mice was similar with *Pd1^fl/fl^* mice. Scale bar, 100 μm. F) Western blot analysis of PDGFRβ in *Pd1^fl/fl^* and *Pd1^cKO-Tie2^* mice brain, β-ACTIN detected as loading control. G) Quantification of PDGFRβ normalized densitometry in western blot. n = 4 mice for each group. two-tailed unpaired t test. H) Wnt related genes were analyzed by RT-qPCR in purified ECs from *Pd1^fl/fl^* and *Pd1^cKO-Tie2^* mice. n = 6 mice, p = 0.00018 (*Lef1*), p = 0.002205 (*Axin2*), p < 0.0001 (*Apccd1*), p = 0.002879 (*Nkd1*), multiple t tests, statistical significance determined using the Holm-Sidak method, with alpha = 0.05. I)The BBB function is normal in adult *Pd1^cKO-Tie2^* mice. Scale bar, 200 μm. Data are means and SEM, two-tailed unpaired t-test, p < 0.05(*), p < 0.01(**), p < 0.001(***), ns not significant.


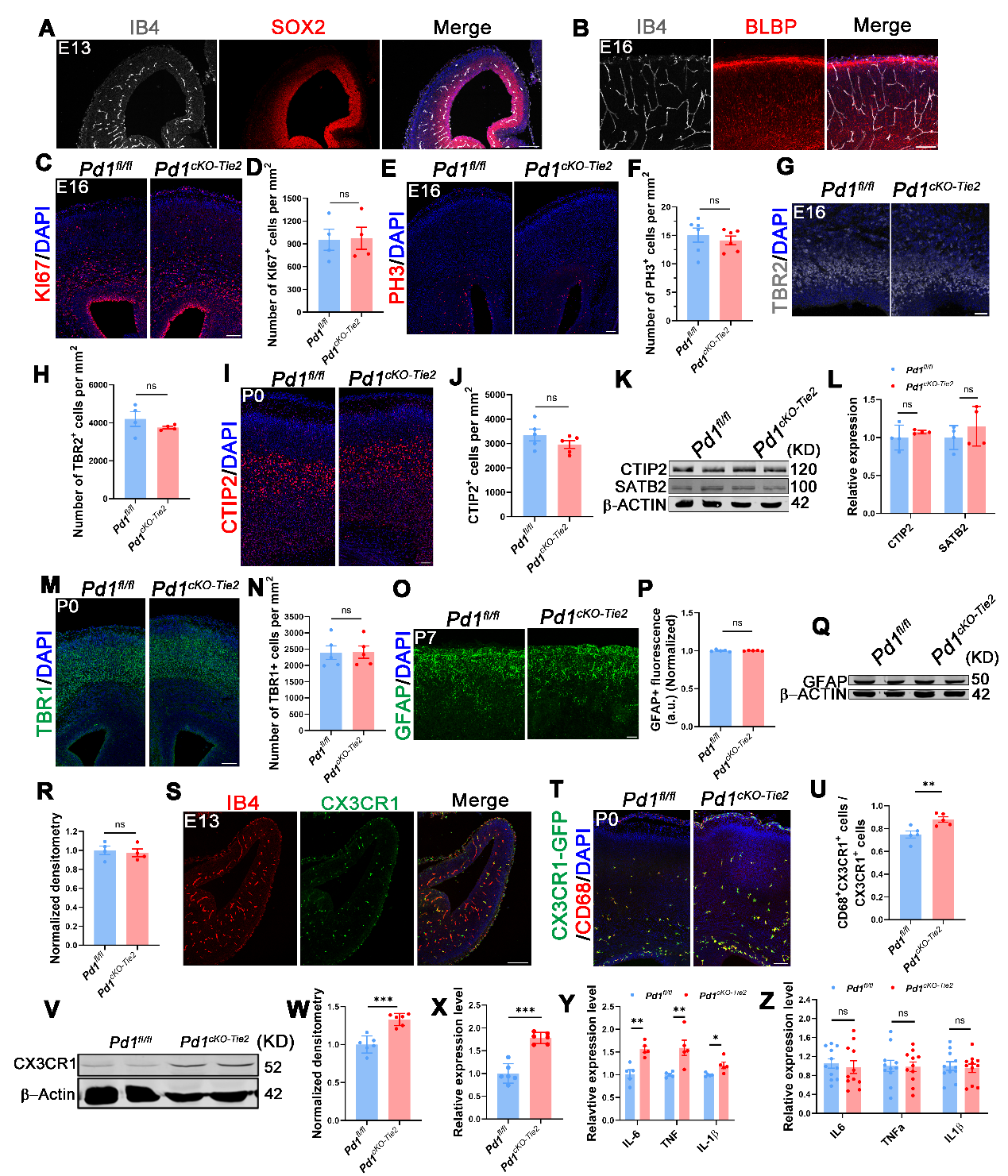


**Figure S3.** Endothelia PD-1 deletion does not affect neurogenesis and astrogenesis but increase brain inflammatory level. A) Immunofluorescence staining of SOX2^+^ neural stem cells and IB4^+^ vessels at E13 cerebral cortex. Scale bars, 100 μm. B) Immunofluorescence staining of BLBP^+^ glia progenitor cell and IB4 vessel^+^ at E16 cerebral cortex. Scale bar, 100 μm. C) The expression of KI67 in *Pd1^cKO-Tie2^* mice was similar with *Pd1^fl/fl^* mice. Scale bar, 100 μm. D) Graph showed the number of KI67 positive cells in E16 cortex. n = 4 mice for each group, two-tailed unpaired t test. E) Mitotic index at E16. Scale bar, 100 μm. F) Graph showed the number of PH3 positive cells in cerebral cortex was similar between *Pd1^fl/fl^* mice and *Pd1^cKO-Tie2^* mice. n = 6 mice for each group, two-tailed unpaired t test. G) Confocal images of TBR2^+^ cells in E16 mice VZ/SVZ zones. Scale bar, 50 μm. H) Graph showed the number of TBR2^+^ cells in VZ/SVZ zones was similar between *Pd1^fl/fl^* mice and *Pd1^cKO-Tie2^* mice. n= 4 mice for each group, two-tailed unpaired t test. I) Confocal images­ of CTIP2^+^ cells at E16 in *Pd1^fl/fl^* mice and *Pd1^cKO-Tie2^* mice cortex, Scale bar, 100 μm. J) Graph showed the number of CTIP2^+^ cells. n = 5 mice for each group, two-tailed unpaired t test. K) Western blot analysis of the expression level of CTIP2 and SATB2 in *Pd1^fl/fl^* mice and *Pd1^cKO-Tie2^* mice cortex. L) Quantification of CTIP2 and SATB2 protein normalized densitometry in western blot. n = 4 mice for each group, multiple t tests, statistical significance determined using the Holm-Sidak method, with alpha = 0.05. M) Confocal immunofluorescence images of TBR1^+^ cells at P0 in *Pd1^fl/fl^* mice and *Pd1^cKO-Tie2^* mice cortex. Scale bar, 100 μm. N) Graph showed the number of TBR1 positive cells in cortex. n = 5 mice for each group, two-tailed unpaired t test. O) Confocal immunofluorescence images of GFAP^+^ cells at P7 in *Pd1^fl/fl^* mice and *Pd1^cKO-Tie2^* mice cortex. Scale bar, 50 μm. P) Graph shows the GFAP normalized fluorescence intensity in cortex. n = 5 mice for each group, two-tailed unpaired t test. Q) Western blot analysis of the expression of GFAP in *Pd1^fl/fl^* and *Pd1^cKO-Tie2^* mice brain, β-ACTIN detected as loading control. R) Quantification of GFAP protein normalized densitometry in western blot, n = 4 mice for each group, two-tailed unpaired t test. S) Immunofluorescence staining of CX3CR1 and IB4 at E13 cerebral cortex. Scale bar, 100 μm. T) Confocal images of CX3CR1-GFP and CD68 in *Pd1^fl/fl^* and *Pd1^cKO-Tie2^* mice brain section at P0. Scale bar, 200 μm. U) The number of the ratio CX3CR1^+^CD68^+^ cells was increased in *Pd1^cKO-Tie2^* mice brain. n = 5 mice for each group, p = 0.0097, two-tailed unpaired t test. (V) Western blot analysis of expression of CX3CR1 in *Pd1^fl/fl^* and *Pd1^cKO-Tie2^* mice cortex, β-ACTIN detected as loading control. (W) Analysis of normalized densitometry of CX3CR1 in western blot. n= 6 mice for each group, p = 0.0002, two-tailed unpaired t test. X) RT-qPCR analysis of relative CX3CR1 mRNA level in the *Pd1^fl/fl^* and *Pd1^cKO-Tie2^* brain. n = 6 mice for each group, p < 0.0001, two-tailed unpaired t test. Y) RT-qPCR analysis showed that the relative IL-6, TNF and IL1β mRNA levels in *Pd1^cKO-Tie2^* mice brain were increased. n = 5 mice for each group, p = 0.001644 (IL-6), p = 0.008394 (TNF), p = 0.020781 (IL-1β), multiple t tests, statistical significance determined using the Holm-Sidak method, with alpha = 0.05. Z) RT-qPCR analysis showed that relative IL-6, TNF and IL1β mRNA level in the E13 *Pd1^fl/fl^* and *Pd1^cKO-Tie2^* mice brain were similar. n = 11 mice for each group, multiple t tests, statistical significance determined using the Holm-Sidak method, with alpha = 0.05. Data are means and S.E.M, p < 0.05(*), p < 0.01(**), p < 0.001(***), ns not significant.


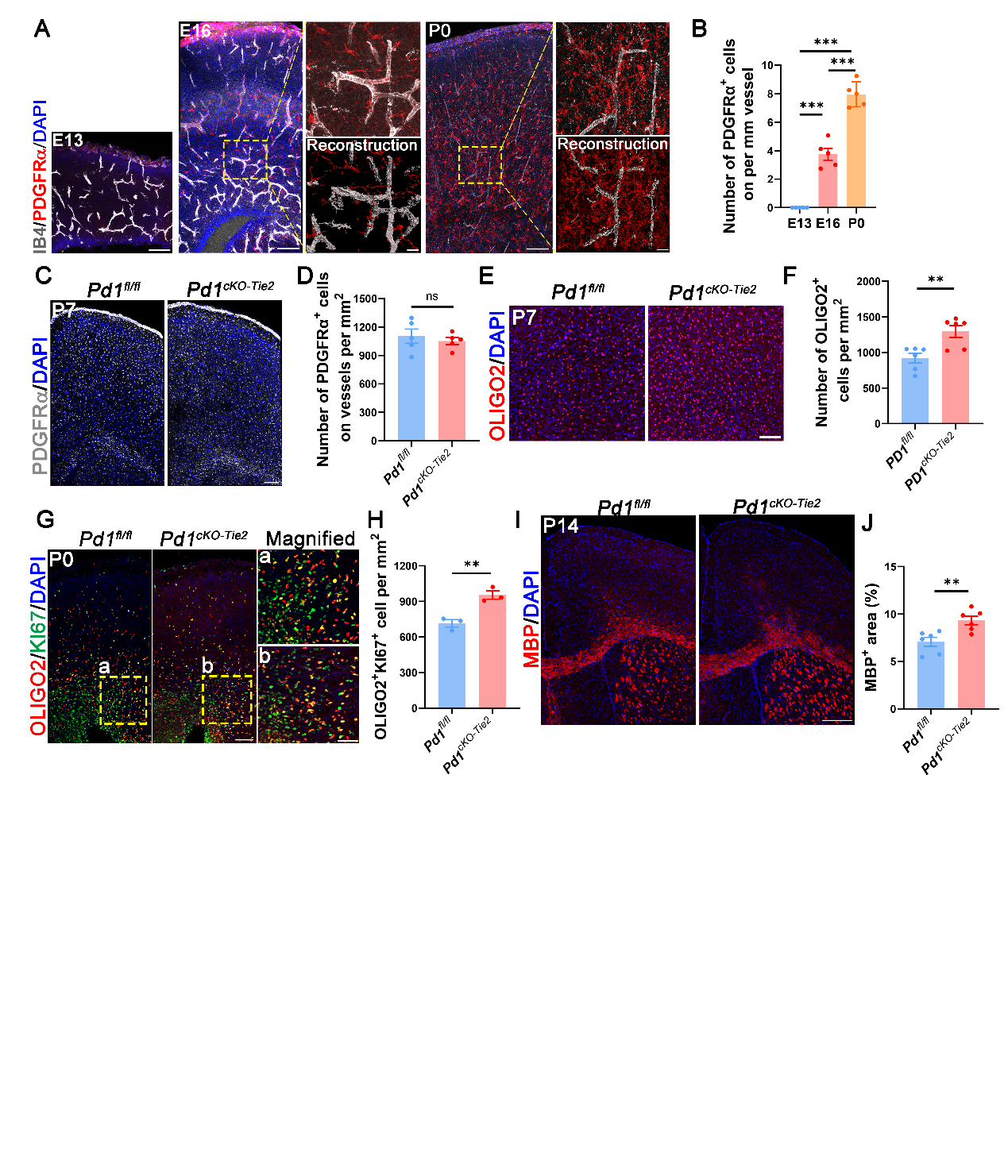


**Figure S4.** Endothelial PD-1 knockout causes OPC hyper-differentiation. A) Immunofluorescence staining of PDGFRα and IB4 at E13, E16, and P0 in the cerebral cortex. Right, the magnified images and the 3D reconstruction images of the delineated area. Scale bars, 100 μm (left), 20 μm (right). B) Graph showed the number of OPC attached to vessel per mm^2^. n = 5 mice for each group, p < 0.0001 (E13 vs. E16), p < 0.0001 (E13 vs. P0), p < 0.0001 (E16 vs. P0). one-way ANOVA with Tukey’s multiple comparison test. C) The number of PDGFRα^+^ cells of P7 *Pd1^cKO-Tie2^* mice were similar to *Pd1^fl/fl^* mice. Scale bar, 100 μm. D) Graph showed the number of PDGFRα^+^ cells in the cortex. n = 5 mice for each group, two-tailed unpaired t test. E) The number of OLIGO2^+^ cells of P7 *Pd1^cKO-Tie2^* mice was increased compared with *Pd1^fl/fl^* mice. Scale bar, 100 μm. F) Graph showed the number of OLIGO2 positive cells per mm^2^ in the *Pd1^fl/fl^* mice and *Pd1^cKO-Tie2^* cortex. n = 6 mice for each group, p = 0.006, two-tailed unpaired t test. G) Coronal brain slices of P0 *Pd1^fl/fl^* and *Pd1^cKO-Tie2^* mice were stained with anti-OLIGO2 and KI67, the right panel showed the magnified image of the delineated area. Scale bar, 100 μm (left), 50 μm (right). H) The number of OLIGO2^+^ KI67^+^ cells in cerebral cortex of *Pd1^cKO-Tie2^* mice was increased. n = 3 mice for each group and p = 0.0067, two-tailed unpaired t test. I) The area of MBP^+^ (%) in cerebral cortex of *Pd1^cKO-Tie2^* mice at P14 was increased. Scale bar, 200 μm. J) Graph represents the area of MBP *^+^* (%) in *Pd1^fl/fl^* and *Pd1^cKO-Tie2^* mice cerebral cortex. n = 6 mice for each group p = 0.0064, two-tailed unpaired t test. Data are means and SEM, p < 0.05(*), p < 0.01(**), p < 0.001(***), ns not significant.


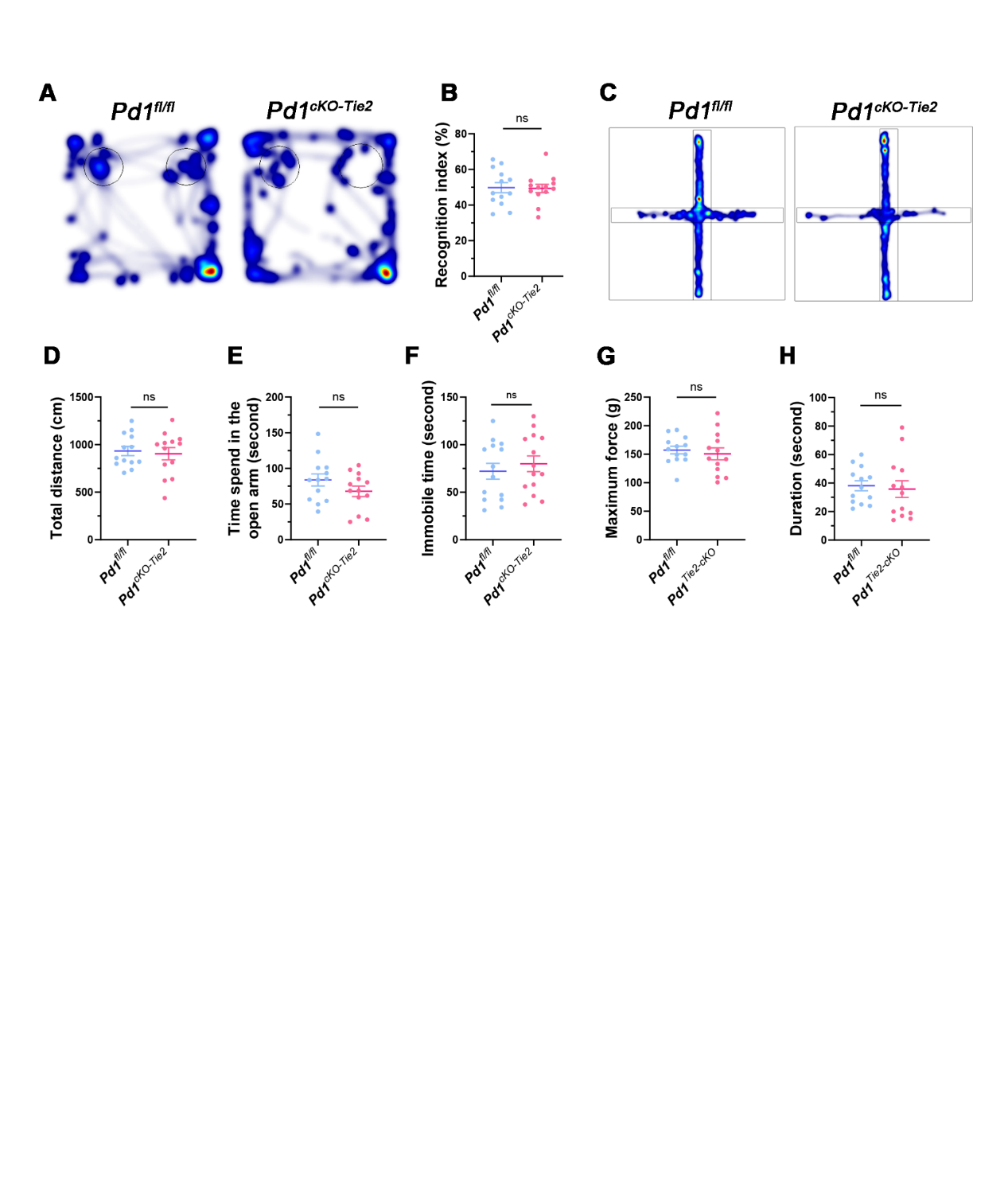


**Figure S5.** Loss of endothelial PD-1 does not lead to depression or anxiety-like behavior. A) Representative images of the novel thing recognition test. B) Graph showed no significant difference between two groups in the novel thing recognition test. n = 13 mice for each group, two-tailed Unpaired t test. C) Representative images of the evaluative cross maze test. D) Graph showed the total distance is similar between two groups in the evaluative cross maze test. n = 13 mice for each group, two-tailed Unpaired t test. E) Graph showed the time spent in open arms was similar between two groups in the evaluative cross maze test. n = 13 mice for each group, two-tailed unpaired t test. F) Graph showed the immobile time was similar between two groups in the forced swim test. n = 14 mice for each group, two-tailed unpaired t test. G) Graph showed the maximum force was similar between two groups in the grip force test. n = 13 mice for each group, two-tailed unpaired t test. H) Graph showed the balanced time spent in the rotarod test was similar between two groups. n = 13 mice for each group, two-tailed unpaired t test. Data are means and SEM, two-tailed unpaired t-test, p < 0.05(*), p < 0.01(**), p < 0.001(***), ns not significant.
